# Inhibition of cued but not executed task sets depends on cue-task compatibility and practice

**DOI:** 10.1007/s00426-024-02013-z

**Published:** 2024-07-30

**Authors:** Alexander Berger, Iring Koch, Markus Kiefer

**Affiliations:** 1 Section for Cognitive Electrophysiology, Department of Psychiatry, Ulm University, Leimgrubenweg 12, 89075 Ulm, Germany; 2 Institute of Psychology, RWTH Aachen University, Aachen, Germany

## Abstract

In task switching, processing a task cue is thought to activate the corresponding task representation (“task set”), thereby allowing for advance task preparation. However, the contribution of preparatory processes to the emergence of n−2 repetition costs as index of task set inhibition processes is debated. The present study investigated whether merely preparing for a task activates a corresponding task set, which needs to be inhibited in order to switch to a different task. To this end, we presented so-called *task cue-only trials* in trial n−2 and assessed subsequent n−2 repetition costs. The results revealed n−2 repetition costs following a task cue-only, but only for compatible cues with a transparent cue-task relation and only at the beginning of the experiment. In contrast, n−2 repetition costs following task execution in trial n−2 were absent. In a second experiment, we sought to rule out that the presence of n−2 repetition costs following a task cue-only and the corresponding absence following task execution were the consequence of a decay of task sets. This second experiment replicated the result pattern of the first experiment, with n−2 repetition costs following a task cue-only being present only at the beginning of the experiment and only for compatible cues. Hence, cue-induced task set inhibition effects depended on cue-task compatibility and practice. Furthermore, merely prepared task sets were more likely inhibited than executed task sets.

**Supplementary Information:**

The online version contains supplementary material available at 10.1007/s00426-024-02013-z.

## Introduction

The flexibility of the human cognitive system is often studied within the context of the task switching paradigm (Monsell, 2003). In this paradigm, participants have to perform at least two tasks and performance is usually impaired if the task switches compared to when it repeats, termed switch costs (Kiesel et al., 2010; Koch et al., 2018; Koch & Kiesel, 2022). In a widely used variant of this experimental procedure, the *cued* task switching paradigm, a task cue indicating the to-be-performed task is presented before the stimulus, therefore allowing in advance task preparation (Jost et al., 2013). Whether and how such preparatory processes contribute to task switching effects is a matter of debate (Jost et al., 2008, 2013; Logan & Bundesen, 2003; Schneider & Logan, 2005; Vandierendonck et al., 2010). The present study therefore elucidates such preparatory processes in task switching in more detail by investigating the involvement of cue-related processing on task set inhibition effects.

### Measuring task set inhibition in task switching

In the context of task switching research, a task set can be defined as the cognitive configuration required for performing a particular task and includes, for example, relevant stimulus dimensions and response categories (Monsell, 2003; Rogers & Monsell, 1995). A common theory of task switching processes, termed *task set reconfiguration*, postulates that when switching tasks, the cognitive configuration needs to be endogenously changed in order to implement the new task set required in the current trial (Kiesel et al., 2010; Monsell, 2003; Vandierendonck et al., 2010). Following this view, the activation of the newly required task set must overcome the activation of the previous task set (when tasks switch) in order to be able to appropriately perform the new task. To this end, an inhibition of the task set (of the previous trial) is commonly assumed (Koch et al., 2010). Often, such inhibition mechanisms are studied using so-called n−2* repetition costs* (Koch et al., 2010; Mayr & Keele, 2000).^1^ n−2 repetition costs are investigated in task switching paradigms using three tasks and are defined as a worse performance in the last task (trial n) in task sequences of the ABA type (i.e. when the task repeats from trial n−2 to trial n) compared to CBA sequences (i.e. when the task switches from trial n−2 to trial n). This performance impairment in ABA sequences compared to CBA sequences is thought to reflect the inhibition of task set A during the transition from task A to B. As task set A was more recently inhibited in sequences ABA compared to CBA (in CBA sequences, the most recent execution of task A can be in trial n−3), it is more demanding to overcome this persisting inhibition in trial n, resulting in the above described performance decrements (Koch et al., 2010).

However, there is a debate regarding the processes contributing to n−2 repetition costs (reviewed in Koch et al., 2010; Vandierendonck et al., 2010). Among these processes, an inhibition of task sets, and episodic interference effects were discussed. A recent study directly tested the contribution of these processes on n−2 repetition costs (Schuch & Keppler, 2022), and found evidence for both top-down driven influences acting on the level of task sets (task set inhibition; see also Mayr, 2002) and more bottom-up, task- or stimulus-related influences (episodic interference; see also Grange, 2018; Grange et al., 2017). Continuing this debate, the present study tried to identify whether the sole activation of a task set is sufficient to produce n−2 repetition costs. Task set activation was realized in the present study by presenting *task cue-only trials*, i.e. task sets are only cued, but not executed as no task stimulus is presented, thereby ruling out any possible influences of task- or stimulus-related processes. Using this procedure, the present work aims to elucidate whether merely cued, but not executed task sets are similarly inhibited in preparation for a new task, as it is assumed for executed task sets. This question is relevant because previous evidence on the role of cue-related and response-related processes in task inhibition revealed inconsistent findings. We review these findings below.

### Influence of preparatory processes on task set inhibition

Several lines of research have addressed the contribution of preparatory processes to n−2 repetition costs, using different approaches to estimate such an influence. As it is unlikely that n−2 repetition costs can be reduced to an inhibition of the cue representation itself (Gade & Koch, 2008), influences of preparatory processes on n−2 repetition costs probably act upon the ease or difficulty of reconfiguring task sets, not simply an encoding of cues. Regarding the detection of such an involvement of task preparation to n−2 repetition costs, studies first varied the cue-target interval (CTI), i.e. the time interval available for cue processing. In task switching with two tasks, switch costs are usually reduced with longer CTIs, indicating that participants use the additional time for cue processing to prepare for a task switch (Jost et al., 2013; Kiesel et al., 2010). For the influence of CTI on n−2 repetition costs, there is less consistency regarding the direction of this influence in the literature. For example, Gade and Koch (2014) provided an overview of published studies reporting the influence of a CTI manipulation on n−2 repetition cots, which showed a diverse pattern with increases and decreases of n−2 repetition costs with longer CTI. This inhomogeneity might be explained by additional factors modulating the influence of CTI on n−2 repetition costs like cue type (Gade & Koch, 2014) and the CTI in trials n−2 and n−1 (Scheil & Kleinsorge, 2014a).

Second, a go/no-go methodology was implemented (Philipp et al., 2007; Scheil & Kleinsorge, 2022; Schuch & Koch, 2003). The rationale was that stimulus processing (or at least response selection and execution) is terminated by the presentation of a no-go signal (the go-/no-go signal was presented either with or after stimulus onset), therefore elucidating to what degree task preparation processes are involved in n−2 repetition costs. In short, these studies showed that response selection and execution processes are relevant for the occurrence of n−2 repetition costs (Philipp et al., 2007; Schuch & Koch, 2003), suggesting that sole task preparation may not be sufficient for inducing task set inhibition (but see Scheil & Kleinsorge, 2022, for conflicting evidence, when no-go trials are bound to only one task).

Third, the influence of task preparation processes on n−2 repetition costs was also investigated by varying the cue itself. By manipulating task cue transparency / cue-task compatibility, i.e. how straightforward the relation of the task cue and associated decision categories is (Jost et al., 2013), it was investigated whether n−2 repetition costs depend on the supposed ease of task set activation in the cue interval. Similar to findings for switch costs (Gade & Steinhauser, 2020; Jost et al., 2013), n−2 repetition costs were reduced with more transparent / compatible cues (Gade & Koch, 2014; Houghton et al., 2009), suggesting a more efficient reconfiguration of task sets if the cue activates the task set more directly and thus activation of the new task is facilitated.

Taken together, these studies showed a rather inconsistent picture regarding the contribution of preparatory processes to task set inhibition indexed by n−2 repetition costs. However, they demonstrated a susceptibility of n−2 repetition costs to manipulations of preparatory stages in the task switching procedure (for a more general overview for the susceptibility of task switching effects to variations of the experimental procedure, see Koch et al., 2018). Moreover, these findings do not unequivocally support the preparatory-related account of n−2 repetition costs, as in all these studies, task cues were always followed by a stimulus. Hence, it is conceivable that stimulus- or task set execution-related processes have affected the above-described effects. To isolate the influence of cue-related processes from stimulus-related or task set execution-related processes, a promising approach is to present so called *task cue-only trials*, that is, task cues that are not followed by a task stimulus and thus also not by an executed task.

In task cue-only trials, only the task cue is shown unpredictably without the presence of a following stimulus, and after a certain interval the next trial follows. Hence, only the task cue can be processed in task cue-only trials. The presence of switch costs in studies using two tasks could be already demonstrated after task cue-only trials (Lenartowicz et al., 2011; Swainson et al., 2021, 2023), indicating that sole task cue-induced task preparation is sufficient to induce a performance deficit in the following trial if the task set switches (see also Kleinsorge et al., 2005; Kleinsorge & Gajewski, 2004).

While the presentation of task cue-only trials hence appears to be a promising method to address the preparatory account of n−2 repetition costs, we are only aware of one study utilizing task cue-only trials in the context of n−2 repetition costs. Prosser et al. (2022) investigated whether the presentation of a task cue-only in trial n−1 is sufficient to trigger an inhibition of the task set performed in trial n−2 (i.e. only trial triplets including a task presented in trial n−2 were analyzed). The results showed a lack of reliable n−2 repetition costs in task responses, similar to studies presenting a no-go signal in trial n−1 (Gade & Koch, 2007; Schuch & Koch, 2003), suggesting that a task cue-only might not be sufficient to trigger an inhibition of a previously performed task set (Prosser et al., 2022). However, to the best of our knowledge, no previous study directly tested whether cue-induced task set activation itself is the target of inhibitory processes in preparation of an upcoming task, i.e. whether task set reconfiguration (and an accordingly assumed inhibition of the previous task set) occurs after task cue-only presentation. To this end, unlike presenting cue-only trials in trial n−1, as Prosser et al. (2022) did, the present study presented task cue-only trials in trial n−2.

### Task set inhibition following task cue-only presentation

Regarding the question whether task sets become inhibited following a task cue-only, previous studies were conducted in our lab in the context of a modulation of masked semantic priming (Berger et al., 2022; Kiefer et al., 2019). These studies were motivated by the attentional sensitization model of unconscious cognition (Kiefer & Martens, 2010), which postulates that activated task sets sensitize subsequent unconscious processing. In line with predictions of this model, it was previously shown that masked semantic priming is enhanced after the execution of a semantic task, indicating that performing the semantic task sensitizes processing pathways also later involved in semantic prime processing (Kiefer, 2019; Kiefer & Martens, 2010; Martens et al., 2011; Ulrich et al., 2014). Following this line of research, in those studies we assessed masked semantic priming in a masked primed lexical decision task subsequent to task cue-only trials associated with a semantic and a perceptual task, to infer from the modulation of priming the preceding activation of task sets following task cue-only presentation. However, in contrast to executed tasks, following the presentation of a task cue-only, semantic priming was mostly larger for perceptual than semantic task sets (Berger et al., 2022; Kiefer et al., 2019). This effect was interpreted in terms of an inhibition of task sets subsequent to task cue-only trials: only prepared, but not executed task sets were assumed to be inhibited (presumably to facilitate performance of the conflicting task set of the masked primed lexical decision task), resulting in the reversed modulation of masked priming compared to that following task set execution.

### Influence of cue-task compatibility on task set inhibition effects

Kiefer et al. (2019) found inhibition of task sets following a task cue-only only if the task cue and associated decision categories matched, suggesting that it depended on properties of the task cue. This manipulation was later called cue-task compatibility (Berger et al., 2022), with task cues matching the decision categories of the task termed compatible cues and non-matching task cues termed incompatible cues. The cue-task compatibility manipulation in Kiefer et al. (2019) was motivated by a previous task switching study (Jost et al., 2017), which showed that a dominant task (strong stimulus-response bindings based on spatially compatible S-R mapping rules) received more inhibition than other, less dominant, tasks. Following this line of reasoning, Kiefer et al. (2019) reasoned that the presence of inhibition effects only following compatible task cues suggests that task sets triggered by compatible task cues are more dominantly represented according to the straightforward cue-task relation. Accordingly, they should be more in conflict with other tasks and receive more inhibition when a different task than the cued task has to be performed. However, a recent study found a comparable level of task set inhibition effects following task cue-only trials for compatible and incompatible task cues (Berger et al., 2022), which may be due to a greater engagement in task preparation for incompatible cues as suggested by electrophysiological recordings (Berger & Kiefer, 2024). This suggests that task sets triggered by both compatible and incompatible task cues might need to be inhibited when switching to another task (at least in the context of a modulation of masked priming). In contrast, studies in task switching with always executed tasks showed a different influence of cue-task compatibility. In these studies, n−2 repetition costs were smaller for compatible than incompatible cues, suggesting stronger task set inhibition effects for incompatible cues (Gade & Koch, 2014; Houghton et al., 2009).^2^

However, these two lines of research differed on a conceptual level. n−2 repetition costs measure the after-effects of task set inhibition in terms of the influence of a previously inhibited task set on performance in trial n. In contrast, our previous studies assessed the influence of a presumably inhibited task set following a task cue-only on masked semantic priming in an immediately following primed lexical decision task. Hence, direct and persisting influences of task set inhibition may dissociate, especially because in task switching studies a new task cue is presented in trial n, and task set activation in task switching was shown to be facilitated with compatible task cues (Jost et al., 2013). In light of the above-outlined influence of cue-task compatibility on task set inhibition effects, the present study was aimed to investigate how task set inhibition effects following a task cue-only observed in the context of a modulation of priming can be extended to task switching and whether this task set inhibition depends on cue-task compatibility.

### Influence of task practice on task set inhibition effects

Besides the influence of cue-task compatibility, our previous work also revealed an influence of task practice upon task cue effects on masked priming (Berger et al., 2022, 2024), indicating that task set activation (and likewise a subsequent inhibition) following a task cue may change with practice. Probably, there is a reduced need for distinct advance task preparation with practice, as task sets can be more easily retrieved with stimulus onset, i.e. when the need for task execution is evident (Berger et al., 2024). An accordingly less pronounced task preparation process following a task cue-only with practice should result in lower demands to inhibit cued task sets. This would be in line with results in task switching, showing a reduction of n−2 repetition costs following task set execution with practice (Grange & Juvina, 2015; Scheil, 2016). This practice effect on n−2 repetition costs was (partially) driven by a reduced influence of task set inhibition processes (Grange et al., 2019). Accordingly, the amount of task practice is a further relevant factor for the investigation of task set inhibition effects, especially following a task cue-only.

### The present study

To study an assumed inhibition of task sets following task cue-only trials, we assessed n−2 repetition costs and tested whether such costs can be observed following a task cue-only, and how such costs are moderated by cue-task compatibility and task practice. To this end, we presented task cue-only trials in a paradigm in which participants had to switch between three tasks in order to assess n−2 repetition costs as an index of task set inhibition processes (Koch et al., 2010). In contrast to previous work (Prosser et al., 2022), task cue-only trials were presented in trial n−2 in order to estimate whether task sets are inhibited following task cue-only presentation, i.e. whether mere task cue presentation is sufficient to trigger a task set activation, which needs to be inhibited when switching to another task. This prediction is based on the assumption that task set inhibition is engaged in preparation of an upcoming task only if the earlier task set had been previously activated and has now to be abandoned.

The amount of task set inhibition triggered by task cue-only trials was compared to that one triggered by task execution, resulting in the following experimental design: In trial n−2, either a task cue followed by a semantic or perceptual classification task or a task cue-only was presented. The task cue-only cued the semantic/perceptual task, for which, however, the actual target stimulus was not presented, so that, accordingly, the task could not be performed. In trial n−1, a lexical decision task (LDT) was presented. Note that this LDT used different stimuli compared to the semantic and perceptual task and was not explicitly cued. This design was chosen to closely resemble our previous work investigating the modulation of masked priming (Berger et al., 2022; Kiefer et al., 2019), which indicated task sets following a task cue-only to be inhibited in order to perform a LDT (where masked priming was assessed). In trial n, a task cue followed by a semantic or perceptual classification task was presented. Accordingly, the (cued or executed) task set could repeat or switch from trial n−2 to trial n, comprising ABA and CBA sequences, which were used for determining n−2 repetition costs. For an overview of the experimental design, see Table 1.

**Table 1:** Overview of the experimental design of the present study

n−2 trial type	Task set sequence	Trial n−2		Trial n−1		Trial n	
		Semantic/perceptual task or task cue-only		Lexical decision task		Semantic/perceptual task	
		Cue	Target	Cue	Target	Cue	Target
Task	*ABA*	R		n/a	Metal	R	
	*CBA*	B		n/a	Sorde	R	
Task cue-only	*ABA*	R	n/a	n/a	Yalmow	R	
	*CBA*	B	n/a	n/a	Garden	R	

The cue “R” represents the perceptual task (round/elongated decision), while the cue “B” represents the semantic task (living/non-living decision). Hence, the chosen example represents the compatible task cue condition. The lexical decision task (word/pseudoword decision) targets are English translations of the German stimulus material. “N/a” symbolizes the absence of a target or cue

Our design differed from the typical measurement of n−2 repetition costs in task switching in two aspects. First, the LDT in trial n−1 was not cued. Second, the LDT used different stimuli (i.e., letter strings) compared to the semantic/perceptual classification task (i.e., pictures). Accordingly, the pictures were “bivalent” stimuli (i.e., affording two different tasks), while the LDT targets were “univalent” and the semantic/perceptual task could not be applied on the LDT stimuli and vice versa. Following previous research, which showed a reduction or absence of n−2 repetition costs if trial n−1 differed in the stimulus format (Sdoia et al., 2022), the number of possible tasks (Scheil & Kleinsorge, 2023b), the response set (Scheil & Kleinsorge, 2023a), or was a task cue-only one (Prosser et al., 2022), the present design may constitute less than optimal conditions for the detection of n−2 repetition costs. However, the responses of all tasks were mapped onto the same two keys (see “Method” section) and the response sets therefore overlapped between tasks. Hence, there was between-task competition in the response set and according to accounts assuming conflict in the response set to be crucial for the occurrence of n−2 repetition costs (Gade & Koch, 2007; Schuch & Koch, 2003), the present design may therefore fulfill the necessary requirements for observing n−2 repetition costs. Moreover, the absence of a cue in trial n−1 may even be beneficial in terms of ruling out alternative explanations, as it is consequently unlikely that a possible detection of n−2 repetition costs following task cue-only trials is the consequence of an inhibition of the cue representation (according to two cues being presented in direct succession). To elaborate on this, if we would have presented a cue in trial n−1 (which was the LDT), a task cue-only trial would have always been followed immediately by another cue, reflecting a cue switch. According to accounts assuming cue switches to be a major determinant of task switching effects (Logan & Bundesen, 2003; Schneider & Logan, 2005), we consider omitting the task cue in trial n−1 increases the likelihood, that possibly observed n−2 repetition costs reflect an inhibition of (aspects of) the cued task set (see also Gade & Koch, 2008).

### Aim of the present study

The aim of the present study was threefold, resulting in the following research questions:

1. Can n−2 repetition costs be observed following a task cue-only in trial n−2? We suppose task cue-only presentation to be sufficient to trigger an activation of task sets, which needs to be inhibited when switching to a new task. Accordingly, we expect to observe n−2 repetition costs following a task cue-only in trial n−2. In addition and in line with previous observations in task switching (for a review, see Koch et al., 2010), we also expect to find n−2 repetition costs following task execution in trial n−2. That is, we suppose both mere task preparation and task execution to trigger an inhibition of task sets reflected by n−2 repetition costs.
2. Does the inhibition of task sets following a task cue-only depend on properties of the task cue, namely cue-task compatibility? Our previous work and earlier observations in task switching suggested that the size of task set inhibition effects depends on properties of the task cue, with however inconsistent findings. Based on previous findings in the task switching literature (Gade & Koch, 2014; Houghton et al., 2009), we expect larger n−2 repetition costs, both following merely cued and executed task sets, for incompatible task cues.
3. Are there any practice-induced changes in n−2 repetition costs following a task cue-only? Previous work suggested a reduced engagement into task preparation following task cue-only trials with practice (Berger et al., 2024), which should result in lower demands for inhibiting only cued, but not executed task sets. Therefore, we would expect n−2 repetition costs following a task cue-only to decrease with experimental duration.^3^

## Experiment 1

### Method

Design and analysis of Experiment 1 was pre-registered (https://osf.io/37brv). Analysis scripts and data were uploaded to a public repository (https://osf.io/ady8t/).

### Participants

N = 55 participants were recruited for Experiment 1. We excluded n = 2 participants for their mean RT exceeding the sample mean RT in the semantic / perceptual classification tasks or the LDT by 2 SDs. The final sample consisted of N = 53 participants. Mean age was 22.58 years (SD = 2.7) and 43 participants were female (81.1%). Of the final sample, n = 26 participants received compatible cues and n = 27 incompatible cues (for a description of compatible and incompatible cues, see the next section).

As pre-registered, the sample size was chosen to achieve a balanced assignment of the stimulus lists to the cue-task compatibility groups (see next section). It was also chosen to be in line with our former study observing an inhibition of task sets following task cue-only trials in the context of masked semantic priming (Berger et al., 2022) due to a lack of previous task switching studies including a presentation of task cue-only trials in trial n−2. Nevertheless, we performed a post-hoc power analysis using the effect size observed for the interaction of task set sequence and n−2 trial type (see the Results of Experiment 1), which revealed a high power for detecting such an effect for Experiment 1 (1 – β = 0.98).

### Tasks and stimuli

Stimuli for the semantic and perceptual classification task as well as the LDT were taken from previous studies (Berger et al., 2022; Kiefer et al., 2019). Stimuli for the semantic and perceptual classification task were 360 grey-scale pictures, depicting animate and inanimate objects. For the semantic classification task, these pictures had to be classified as depicting a living (e.g. tiger) or a non-living (e.g. knife) object, while pictures had to be classified as a round (e.g. ball) vs. elongated (e.g. pencil) object for the perceptual classification task. The pictures were evenly distributed across the semantic and perceptual classification task (each n = 180). It was ensured that the distribution of the other decision category (e.g. round and elongated objects across the living and non-living objects) was approximately balanced, *χ*^2^(1) = 1.52, *p* = .218. In the LDT, letter strings had to be classified as meaningful German word or as pronounceable, but meaningless pseudoword. There were n = 120 words and n = 120 pseudowords. Length of pseudowords (on average 5.13 letters) and words (on average 5.05 letters) did not differ significantly, *t*(232) = 0.60, *p* = .552.

Participants responded with the left and right button of a response box. The living, round and word decisions were mapped to one button, which participants were instructed to press with their index finger. The non-living, elongated and pseudoword decisions were mapped to the other button, which participants had to press with their middle finger. Right-handers and left-handers responded with the index and middle finger of their dominant hand and the mapping of response buttons was accordingly reversed for left- and right-handers (right-handers: living, round and word decision mapped to the left button; left-handers: living, round and word decision mapped to the right button).

We created 24 different stimulus lists, in which the assignment of pictures and LDT stimuli to the different conditions (see the “Design” section) was counterbalanced. All conditions and response categories were balanced. Furthermore, for each participant, we pseudo-randomized the stimulus list to ensure that all type of trial triplets / conditions were about equally often followed by all trial triplets / conditions, so that participants could not predict which condition would follow (corresponding chi-squared tests did not indicate a significant imbalance between the sequence of conditions, *χ*^2^(49) < 10, *p* > .99, for each stimulus list). Stimulus lists were repeated across participants (each at least 2 times), as we collected more than 24 participants.

Task cues to indicate the semantic and perceptual classification task were the capital letters “R” and “B” as in previous work (Berger et al., 2022). Task cues could either be compatible or incompatible with the cued decision categories by a match or mismatch with the first letter of the first alternative of the decision category. For compatible task cues, the letter “R” indicated the perceptual decision (“*r*und / länglich”, engl. “round / elongated” decision) and the letter “B” the semantic decision (“*b*elebt / unbelebt”, engl. “living / non-living”). For incompatible cues, this assignment was reversed, i.e. the task cue “R” indicated the semantic decision, while the task cue “B” indicated the perceptual decision.

LDT targets, task cues (both in white font), and pictures were presented against a black background at the center of the screen. Pictures had a subtended 3.2 × 3.8 degrees visual angle, and the height of all text stimuli (task cues, LDT targets, feedback) was set to 0.88 degrees visual angle. Participants were seated in frontal of a computer screen at a distance of 70cm. The screen was an LCD device and had a resolution of 1280 × 1024 pixels and a screen refresh rate of 60Hz.

### Procedure

**Fig. 1:**
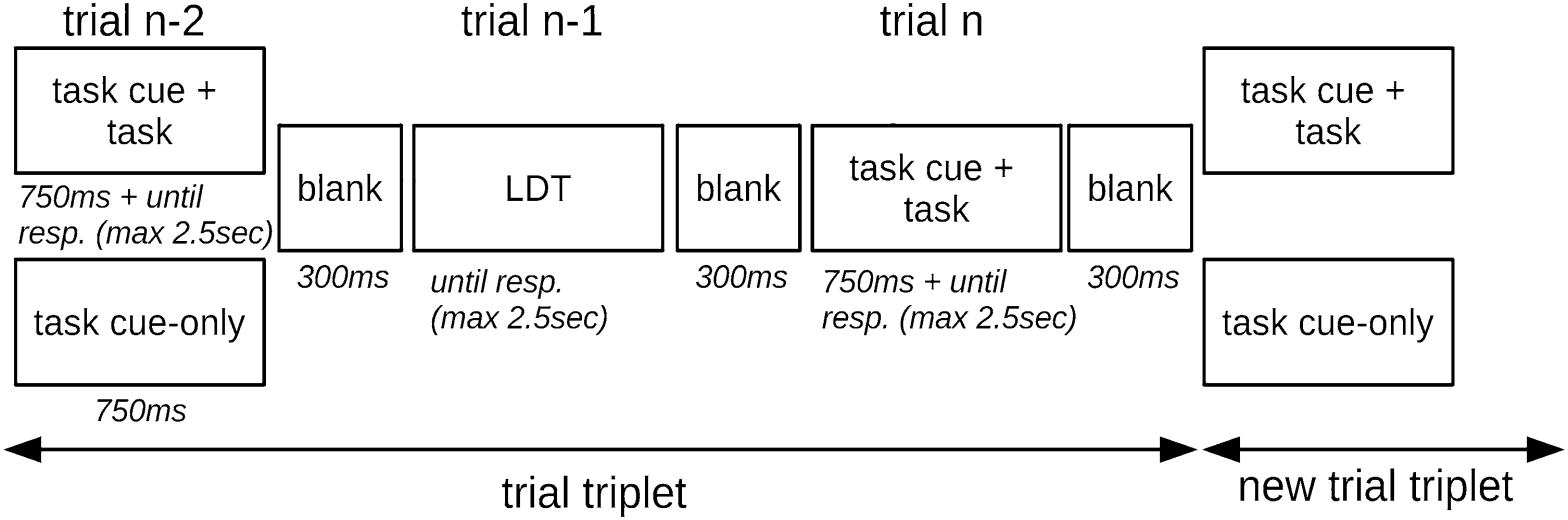
Schematic overview of the experimental procedure in Experiment 1. The experiment was divided into trial triplets. Trial n−2 could either be a task (task cue + semantic or perceptual task) or a task cue-only (the semantic / perceptual task was only cued, but not presented), trial n−1 was a lexical decision task (LDT), and trial n was a perceptual or semantic task. After each trial a blank followed and each trial triplet was immediately followed by the next trial triplet to produce a continuous flow for the participants. Presentation durations are shown in *italic;* “max 2.5sec” refers to the response deadline, which was 2,500ms for each task. The semantic or perceptual task set could either repeat or switch from trial n−2 to trial n, resulting in sequences ABA or CBA, respectively

The experiment was programmed and run in *PsychoPy* (Peirce et al., 2019), version v.2021.2.3. A schematic overview of the experimental procedure is depicted in Fig. 1. The experiment consisted of 720 trials (task cue and task, task cue-only or LDT), which were divided into 240 trial triplets. In the first trial (trial n−2), either a task cue (750ms) and task picture or a task cue-only was presented. The picture immediately followed the task cue and was presented until a response was given or a response deadline of 2500 ms passed. Presentation duration of task cue-only trials was 750 ms as well. After a 300 ms blank, the LDT target was presented in trial n−1, which remained on the screen until a response was made or 2500 ms (response deadline) passed. In trial n, at the end of each trial triplet, after a 300 ms blank a task cue and task followed with the same stimulation properties as in trial n−2. After an additional 300 ms blank, the next trial triplet started, i.e. there was no pause between trial triplets to achieve a continuous flow for the participants. When a response deadline elapsed, feedback (“Zu langsam!”, engl. “Too slow!”) was provided in red for 500ms. The experiment was divided into 8 blocks, and participants could take a break between blocks.

Before the main experiment started, participants practiced the experimental procedure. First, they practiced the LDT, as well as the semantic and perceptual classification tasks in separate blocks. Afterwards, they practiced the semantic and perceptual classification task conjointly in a block. Finally, they practiced the procedure as presented in the main experiment. During practice, participants received feedback about the correctness of their response.

### Design

For the analysis of n−2 repetition costs, there were three factors of interest: Task set sequence, n−2 trial type and cue-task compatibility. Task set sequence and n−2 trial type were manipulated within-subjects, while cue-task compatibility was manipulated between-subjects. Task set sequence had two levels, ABA and CBA, i.e. whether the (merely cued or executed) task set repeated (ABA) or switched (CBA) from trial n−2 to trial n.^4^ n−2 trial type had two levels as well, trial n−2 being a task or being a task cue-only one. Finally, one group of participants received compatible, while the other group received incompatible task cues (see “Tasks and stimuli” section), resulting in a 2 × 2 × 2 mixed design. For each combination of the within-subjects factors, i.e. task set sequences depending on n−2 trial type, 60 trial triplets were presented (240 / 4 = 60).

Furthermore, the experimental procedure allowed us to also analyze task switches and task repetitions, i.e. whether the task from trial n to trial n−2 of the next task triplet switched or was repeated (when trial n−2 of the next trial triplet was a task). Accordingly assessed switch costs could serve as a kind of treatment check, as they constitute a quite stable effect in task switching (Kiesel et al., 2010; Monsell, 2003). For the analysis of switch costs, there were two factors of interest; task sequence (task switch vs. task repetition) and cue-task compatibility, resulting in a 2 × 2 mixed design.

### Statistical analysis

#### Conventional analysis of mean response times and error rates

Single-trial data were pre-processed using the statistical software *R* (R Core Team, 2020). RT outliers were specified separately per trials (task in trial n−2, LDT in trial n−1, task in trial n), but collapsed across task sets as RTs exceeding ± 2 SDs of the individuals mean RT in that task (Berger & Kiefer, 2021, 2023). RT outliers and trials, where no response occurred until the RT deadline, were excluded from all analyses (8.0% of all trials). For the analysis of RTs, we also excluded incorrect responses. Furthermore, for the analysis of n−2 repetition costs, trial triplets with an error, a RT outlier or no response until the deadline in trial n−2 or trial n−1 were excluded as well. After pre-processing, there was an average number of 50.9 (SD = 3.5) per-condition trials available for the analysis of n−2 repetition costs for RTs, and 52.4 (SD = 3.1) trials for ERs.

For conventional analysis of n−2 repetition costs, mean RTs and ERs in trial n were calculated and analyzed using a repeated measures ANOVA with the within-subjects factors task set sequence and n−2 trial type as well as the between-subjects factor cue-task compatibility. For the analysis of task switch costs, a repeated measures ANOVA on mean RTs and ERs in trial n−2 (trial n−2 being a task) was calculated with the within-subject factor task sequence and the between-subjects factor cue-task compatibility. ANOVA analyses were performed using *JASP* (JASP Team, 2020). We report in the “Results” section only summarized results covering the theoretical relevant effects, but the corresponding tables displaying all tested effects can be found in *Supplementary Material E*.

#### Effect course analysis

To investigate how n−2 repetition costs following a task cue-only in trial n−2 develop over the course of the experiment, we calculated effect course analyses. We will only shortly summarize this method, for more detailed information we refer the reader to Berger et al. (2024). Performance (RT and correctness of the response) of trial n in trial triplets with a task cue-only in trial n−2 were extracted for each participant and stored into two separate vectors depending on the task set sequence (ABA vs. CBA). Within these vectors, performance measures were sorted according to their temporal occurrence in the experiment. These vectors were smoothed using moving averages to account for the large variability in single-trial RTs / responses (for further details, see below). Afterwards, the vectors were contrasted to display how the difference between ABA vs. CBA conditions (n−2 repetition costs) develops throughout the course of the experiment. That is, the first occurrence of condition ABA was contrasted against the first occurrence of condition CBA, and so on. To test whether the effect is significant at any time point within the experiment, cluster based permutation testing was applied (CBPT, cf. Maris & Oostenveld, 2007). At each time point / trial (called sample in the context of CBPTs), a dependent t-test was conducted and adjacent samples, in which the t-test crossed an a-priori specified threshold, were grouped together into clusters. Clusters were described by a cluster *T* value, that is the sum of all t-values of the significant tests at the sample level included in this cluster. Significance of clusters was assessed by a permutation procedure, that is in each iteration, separately per participant, the condition assignment was randomly shuffled. In these random permutations of the data, clusters were built as described above. The *p*-value of an observed cluster was defined as the proportion of permutations with a cluster *T*-value exceeding the *T*-value of the observed cluster. For the present analysis, the window size for the calculation of moving averages was set to 13 (60 / 5 + 1), alpha at the sample level was set to α = 0.1 and 5000 permutations were ran comparable to previous work (Berger et al., 2024).

#### Supplementary analyses

Besides the above-mentioned analyses, we performed additional analyses as pre-registered. First, we analyzed whether the performance in the LDT in trial n−1 depended on n−2 trial type, i.e. how switching from a task or a task cue-only to the LDT influenced LDT performance (Supplementary Material B). Furthermore, for this and all other analyses described above, we also performed drift-diffusion model (DDM) analyses (Ratcliff & McKoon, 2008; Voss et al., 2013; Wiecki et al., 2013), which are reported in Supplementary Material A, C, D.

### Results

#### Switch costs in trial n−2

For the analysis of RTs in executed tasks in trial n−2 depending on whether the task switched or repeated from the previous trial triplet, we observed a significant effect of task sequence, *F*(1, 51) = 10.73, *p* = .002, task switches were significantly slower than task repetitions (mean switch costs = 27 ms). Furthermore, the main effect of cue-task compatibility was significant with on average 103ms slower responses for incompatible task cues, *F*(1, 51) = 11.15, *p* = .002. There was also a significant interaction of task sequence × cue-task compatibility (*F*(1, 51) = 10.61, *p* = .002), indicating larger switch costs for incompatible (55 ms) than compatible cues (0 ms), for which switch costs were numerically absent.

Concerning the analysis of ERs, there were numerical switch costs of 0.7%, which however were not statistically significant, *F*(1, 51) = 2.91, *p* = .094. Neither the effect of cue-task compatibility nor the interaction of cue-task compatibility and task sequence reached significance (all *F*s < 1.94. all *p*s > .170).

### n−2* repetition costs*

**Fig. 2:**
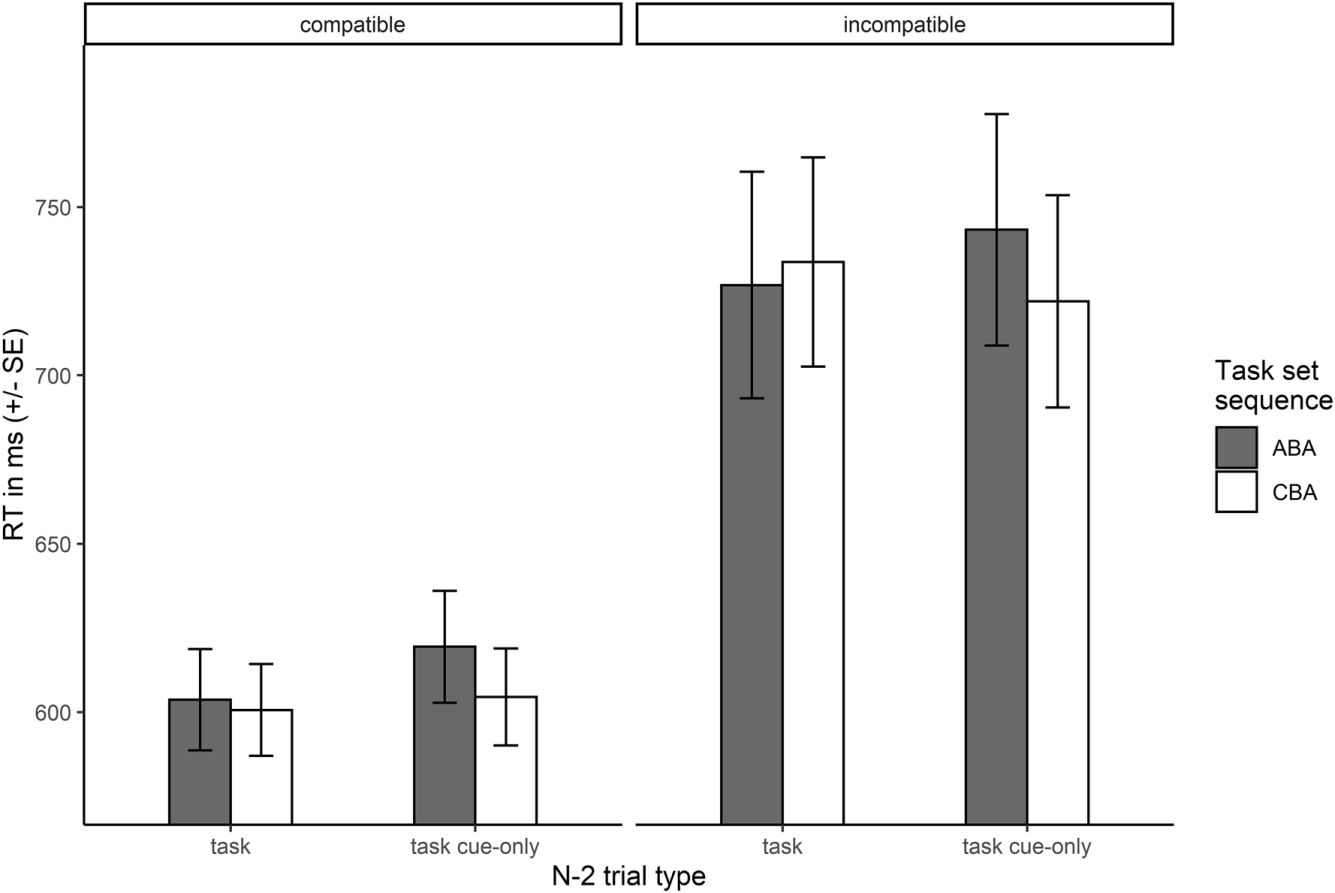
Response times in trial n in Experiment 1. Shown are mean RTs (± SE) in ms depending on n−2 trial type and task set sequence, separately for compatible and incompatible cues

Mean RTs of task performance in trial n are depicted in Fig. 2. As can been seen in this figure, there was a large main effect of cue-task compatibility, *F*(1, 51) = 12.44, *p* < .001, RTs for trials with incompatible task cues were about 124ms slower compared to compatible task cues. Both the main effect of task set sequence (*F*(1, 51) = 2.36, *p* = .130) and n−2 trial type (*F*(1, 51) = 1.20, *p* = .279) were not significant, but there was a significant interaction, *F*(1, 51) = 4.09, *p* = .048, indicating that the effect of task set sequence was modulated by n−2 trial type. Specifically, we observed n−2 repetition costs (18 ms) only following a task cue-only in trial n−2, whereas no n−2 repetition costs were observed following task set execution in trial n−2 (−2 ms). All other interactions did not reach significance, all *F*s < 0.69, all *p*s > 0.41.

A corresponding analysis of ERs in trial n revealed no significant effects, all *F*s < 2.59, all *p*s > .113. Mean ERs as well as mean RTs in trial n depending on task set sequence, n−2 trial type and cue-task compatibility are reported in Table 2.

**Table 2:** Mean RTs and ERs in trial n in Experiment 1

Cue-task compatibility	n−2 trial type	Task set sequence	RT	ER
Compatible	Task	ABA	604 (15)	0.026 (0.004)
		CBA	601 (14)	0.024 (0.006)
	Task cue-only	ABA	619 (17)	0.025 (0.006)
		CBA	604 (14)	0.030 (0.005)
Incompatible	Task	ABA	727 (34)	0.027 (0.005)
		CBA	734 (31)	0.029 (0.005)
	Task cue-only	ABA	743 (34)	0.037 (0.007)
		CBA	722 (32)	0.033 (0.006)

Mean RTs are given in milliseconds, mean ERs in proportion of errors. Associated standard errors are shown in parentheses

#### Effect course analysis of n−2 repetition costs

**Fig. 3:**
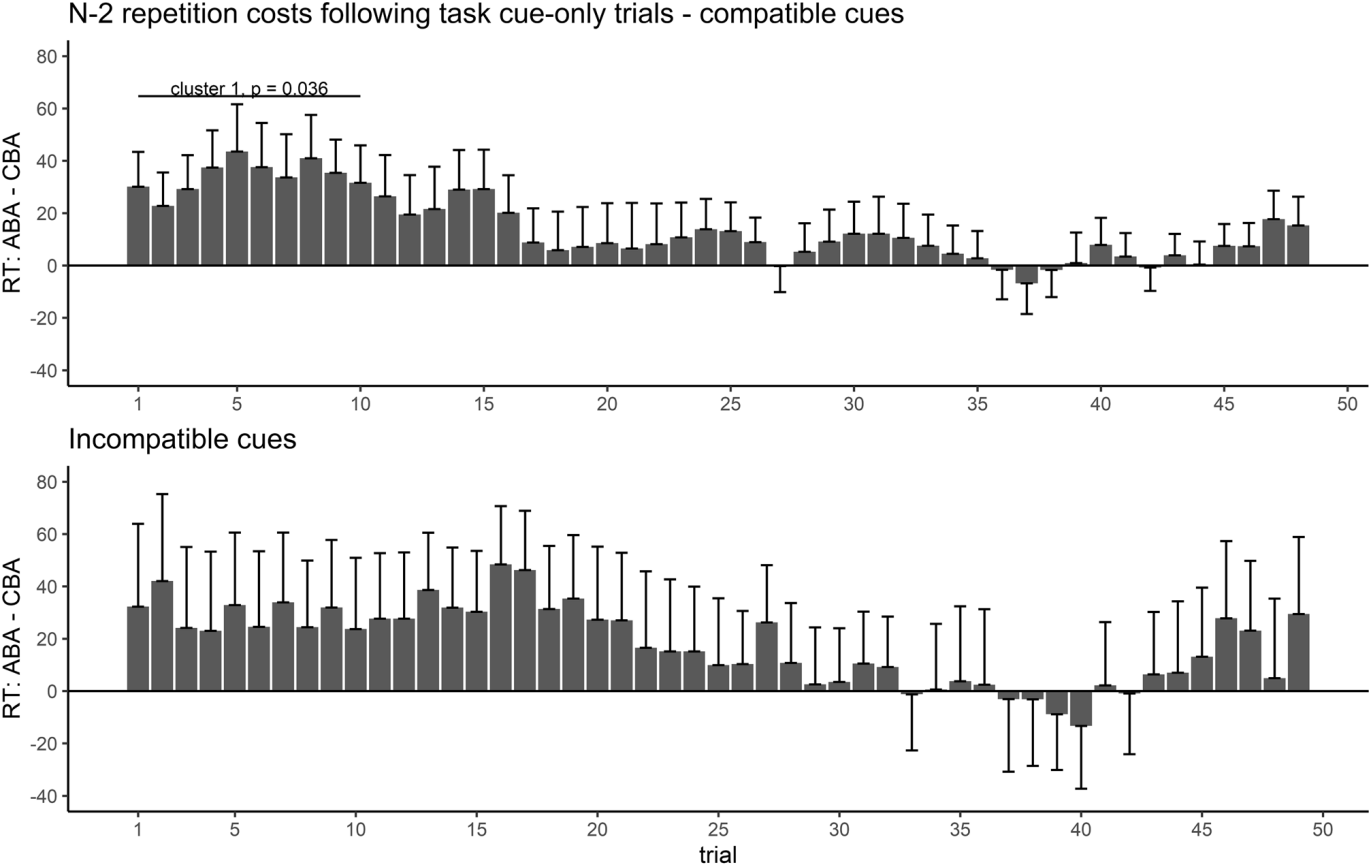
Development of response time n−2 repetition costs following a task cue-only in trial n−2 during the course of Experiment 1. Positive values indicate n−2 repetition costs, negative values n−2 repetition benefits. For both compatible and incompatible task cues, n−2 repetition costs following a task cue-only decreased throughout the experiment. Only for compatible cues, there was a significant cluster at the beginning of the experiment representing reliable n−2 repetition costs

We performed effect course analyses on RTs to assess how the observed n−2 repetition costs following a task cue-only in trial n−2 developed throughout the experiment, i.e. how inhibition effects of only cued, but not executed task sets, changed with practice. The time courses of n−2 repetition costs following a task cue-only, separately for the two cue-task compatibility groups, are depicted in Fig. 3. For both cue types, n−2 repetition costs were maximal at the beginning of the experiment and decreased with increasing experimental duration. For compatible cues, the effect course analysis revealed a significant cluster, ranging from trial 1–10, cluster *T* = 23.06, *p* = .036. Hence, presentation of a task cue-only elicited significant n−2 repetition costs, but only at the beginning of the experiment and only for compatible cues. In contrast, for incompatible cues, while the descriptive pattern was comparable, no significant cluster was observed.

### Discussion

First, as kind of a treatment check, we observed significant switch costs in RTs, i.e. slower responses if trial n of the previous trial triplet was the same task as in trial n−2. These switch costs were only observed for incompatible cues, being in line with previous observations of larger switch costs for incompatible / less transparent cues (Arbuthnott & Woodward, 2002; Gade & Steinhauser, 2020; Grange & Houghton, 2010; Jost et al., 2013). Accordingly, switching tasks produced a cost, predominantly for incompatible cues, which would be typically expected in a task switching experiment (Kiesel et al., 2010; Monsell, 2003). Note that while there was no RT difference between task switches and task repetitions for compatible cues, additional drift-diffusion model analyses indicated numerically a switch cost on drift rate, i.e. processing speed for both cue types (see the Supplementary Material A).

Regarding our research questions, we observed n−2 repetition costs, but only following a task cue-only and not following task execution in trial n−2. Effect course analysis showed n−2 repetition costs following a task cue-only to decrease during the course of the experiment and only for compatible cues, a significant cluster was observed. However, analyses on averaged RTs did not reveal a significant modulation of the n−2 trial type × task set sequence interaction by cue-task compatibility, *F*(1, 51) = 0.68, *p* = .415. Hence, mean n−2 repetition costs following a task cue-only did not differ significantly between compatible and incompatible cues. In contrast, the effect course analysis incorporating a fine-grained resolution of the effect’s development failed to detect a significant cluster for incompatible cues, despite of a similar shape of the effect course compared to compatible cues, probably due to an overall increased variability for incompatible cues. For a more detailed discussion of how n−2 repetition costs developed throughout the experiment and the influence of cue-task compatibility on n−2 repetition costs, see the “General discussion” section.

However, the results of Experiment 1 revealed an unexpected finding that made the interpretation of n−2 repetition costs somewhat difficult. While we observed n−2 repetition costs following a task cue-only, no n−2 repetition costs were present following task (set) execution in trial n−2 (additional drift-diffusion model analyses even indicated an n−2 repetition *benefit* following task execution, see Supplementary Material D). That is, in contrast to usually observed effects in task switching (Koch et al., 2010), we did not observe a cost of repeating the *execution* of the same task set from trial n−2 to trial n. Task preparation is commonly assumed to only constitute one aspect of multiple processes contributing to the occurrence of n−2 repetition costs (Vandierendonck et al., 2010). Hence, this lack of n−2 repetition costs following executed tasks is especially surprising as in task execution the additional influence of response-related processes and episodic interference should add to preparatory influences on n−2 repetition costs (Philipp et al., 2007; Schuch & Keppler, 2022; Schuch & Koch, 2003).

Moreover, the interpretation of n−2 repetition costs following a task cue-only and the absence of such costs following task execution is additionally challenged by a different total duration of both n−2 trial types. In Experiment 1, we equated the blank interval following both trial types, that is following the response to a task and following a task cue-only. For both trial types, there was a blank interval of 300 ms until trial n−1 (the LDT) started. Consequently, the interval from trial onset, i.e. task cue onset, to the next trial was drastically shorter if the n−2 trial type was a task cue-only one. For task cue-only trials, the duration from trial n−2 onset to trial n−1 onset was fixed and lasted 1050 ms (750 ms task cue-only + 300 ms blank interval = 1050 ms), while the corresponding duration for a task in trial n−2 varied from trial to trial and was remarkably longer according to the varying RT of task executions in trial n−2. The mean RT of tasks in trial n−2 was 674 ms, i.e. the average duration of this trial type was approximately 674 ms longer compared to that of task cue-only trials (750 ms task cue + 674 ms response + 300 ms = 1724 ms), while furthermore changing every trial. According to accounts assuming a decay of task sets with time (Altmann, 2002; Altmann & Gray, 2002; Vandierendonck et al., 2010), task set activation following an executed task could have vanished until the onset of the LDT (if one assumes task set activation to start to decay before the response is given), therefore reducing the need for inhibiting that task set (cf. Gade & Koch, 2005). In contrast, as the onset of the LDT followed more shortly after a task cue-only, the strength of the prepared task set may have been stronger, thereby increasing the conflict with the following lexical task set of the LDT, finally resulting in strong demands to inhibit only cued task sets.

However, there could be an alternative explanation for the presence of n−2 repetition costs following a task cue-only and the absence of such costs following task execution: Only cued, but not executed task sets could simply require a larger demand for inhibition, as they interfere to a stronger degree with conflicting task sets. Regarding this aspect, note that previous studies found larger switch costs following task cue-only trials compared to task execution when the preparation interval was short (Lenartowicz et al., 2011; Swainson et al., 2017, 2021). Especially in situations where the lack of task execution following a task cue-only may be surprising, cued but not executed task sets may interfere strongly with conflicting task sets of upcoming tasks and accordingly need to be inhibited. Note that in our experiment, although there was an equal number of task and task cue-only n−2 trial types, 75% of all presented task cues were followed by a task because task cues were always followed by a task in trial n. Hence, if greater inhibition requirements for task cue-only trials compared to task trials is the relevant factor for the differential n−2 repetition costs for these n−2 trial types, we should also expect a similar pattern of n−2 repetition costs when the total duration of both trial types is comparable. To this end, we conducted Experiment 2, in which we aimed to equate the duration of task cue-only and task n−2 trial types to examine whether a different temporal length of these two trial types may have caused the presence of n−2 repetition costs following a task cue-only and the corresponding absence of these costs following executed tasks in Experiment 1.

## Experiment 2

In contrast to Experiment 1, where the blank interval following the response to the task and the offset of the task cue-only in trial n−2 was equated, Experiment 2 equated the total duration of both trial types. That is, for both task execution and mere task preparation (task cue-only), the duration varied from trial to trial and was comparable across trial types. Hence, Experiment 2 tested whether the lack of n−2 repetition costs following task execution in Experiment 1 was the consequence of a decay of task sets. Due to a comparable total duration of task and task cue-only n−2 trial types in Experiment 2, a decay of task sets should similarly affect both trial types, and we would consequently expect to observe a lack of n−2 repetition costs in both n−2 trial types. However, in contrast, if only cued, but not executed task sets require a larger amount of inhibition compared to executed task sets, n−2 repetition costs should remain larger following a task cue-only compared to an executed task. We hypothesized to observe n−2 repetition costs following a task cue-only and a corresponding lack of n−2 repetition costs following task execution, which is a comparable result pattern as in Experiment 1.

### Method

Design and analysis of Experiment 2 was pre-registered (https://osf.io/vkzp8) and analysis scripts and data were uploaded to a public repository (https://osf.io/ady8t/).

#### Participants

For Experiment 2, we recruited a total number of 57 participants. N = 4 participants were excluded from data analysis according to their mean RT in one of the tasks exceeding ±2 SD of the samples mean RT. The final sample consisted of N = 53 participants, mean age = 24.17 (SD = 4.7) years, 37 participants were female (69.8%). Among the participants available for analysis, n = 25 received compatible and n = 28 received incompatible task cues.

As pre-registered, this sample size was chosen to be sufficient to detect a similar modulation of n−2 repetition costs by n−2 trial type as in Experiment 1. A corresponding power analysis revealed a sample size of N = 48 to be sufficient to detect such an effect with a power 1 – β = 0.95 on a significance level of α = 0.05.

#### Procedure

The procedure, tasks, stimuli and design were identical to Experiment 1 except for one aspect: the blank interval following a task cue-only in trial n−2 was prolonged to achieve a comparable duration compared to the interval, when in trial n−2 a task was presented and executed. To this end, the duration of the blank interval following a task cue-only was calculated according to the median RT of that participant for task executions in trial n−2, collapsed across the semantic and perceptual tasks to obtain a larger number of RTs for the calculation of the average RT. Following formula was used for calculating the blank interval following a task cue-only in trial j for a particular participant:

$${Blank}_{Task cue-only,j}=Median\left({RT}_{Task \,trial\, n-2, 1:j}\right)+RNorm\left(\text{0,1}\right) \times SD({RT}_{Task\, trial\, n-2, 1:j})$$

with *Median(RT*_*Task trial n*−2*, 1:j*_*)* representing the median of all RTs collected in tasks in trial n−2 until trial j; *RNorm(0,1)* representing random draws of a standard normal distribution and *SD(RT*_*Task trial n*−2*, 1:j*_*)* representing the standard deviation of all RTs collected in tasks in trial n−2 until trial j.

As this formula requires a sufficient number of RTs collected in trial n−2, it was only applied if at least five RTs were available. Beforehand, the duration of the blank interval following a task cue-only was fixed to 700 ms, i.e. approximately the mean RT of task executions in trial n−2 in Experiment 1. If the formula produced a negative value, the result was multiplied by −1. Following this interval, which approximates the RT in executed tasks in trial n−2, an additional blank of 300 ms followed to ensure a comparable total duration for both n−2 trial types. Note that this procedure might not result in the exact same distribution for RTs in executed tasks and the blank intervals following a task cue-only in trial n−2, as RT distributions are typically not normally distributed (Berger & Kiefer, 2021; Cousineau & Chartier, 2010; Ratcliff, 1979; Whelan, 2008). Nevertheless, as the parameters of the empirical RT distribution of one participant cannot be known to the experimenter in advance of the experiment, an approach had to be taken to mimic that empirical RT distribution. The approach utilized in Experiment 2 fulfills two important prerequisites, which we consider relevant to achieve durations subjectively indistinct from task execution: the blank duration for a participant was calculated based on the RT of that participant and the blank duration varied from trial to trial.

### Statistical analysis

#### Conventional analysis of mean response times and error rates

Pre-processing of RTs and ERs was the same as in Experiment 1. During pre-processing, 7.3% of all trials were excluded as no response occurred until the deadline or the RT was classified as outlier. For the analysis of n−2 repetition costs, there was an average per-condition number of 52.0 (SD = 3.6) available trials with correct responses for the analysis of RTs, and a number of 53.5 (SD = 3.2) trials for the analysis of ERs. Conventional analysis of RTs and ERs using ANOVAs included the same factors as in Experiment 1. The “Results” section only shows summarized results, but corresponding tables including all effects are shown in Supplementary Material F.

#### n−2 repetition costs depending on the blank interval following a task cue-only

Besides conventional analysis of n−2 repetition costs, we also tested whether n−2 repetition costs following a task cue-only depended on the duration of the blank interval. If a decay of task sets would have been the reason for the absence of n−2 repetition costs following task execution in Experiment 1, one would expect n−2 repetition costs to be larger if the blank interval following a task cue-only was shorter, i.e. if the LDT followed the task cue-only more rapidly. To test this hypothesis, we conducted two types of analyses:

1. We analyzed if the duration of the blank interval following a task cue-only in trial n−2 predicts the size of the n−2 repetition costs in trial n. Therefore, we performed a linear mixed model (LMM) analysis on RTs collected in trial n of trial triplets including a task cue-only in trial n−2 (Bates et al., 2014; Kuznetsova et al., 2017). The LMM included subjects as random intercepts and the fixed factors task set sequence, duration of the blank interval and cue-task compatibility as well as all corresponding interactions. If model fit was satisfactory, we also included the fixed factors being manipulated within-subjects (task set sequence, duration of the blank interval) as random slopes. For a corresponding analysis of ERs, we estimated a generalized linear mixed model (GLMM) on the accuracy of the response using a binomial link function. The predictors were coded as follows:
  Cue-task compatibility: compatible cues = 0.5, incompatible cues = −0.5
  Task set sequence: ABA = 0.5, CBA = −0.5
  Duration of the blank interval following a task-cue only: this continuous predictor was z-standardized to ensure that its scale was comparable to that of the other predictors.
  For the analysis of RTs, the LMM included the duration of the blank interval as a random slope. However, for the analysis of accuracies, incorporating any random slope resulted in poor model fit; consequently, this model included only Subjects as a random intercept.
2. We also tested whether a participant’s mean duration of the blank interval being relatively short or long compared to the sample’s mean duration affects the size of n−2 repetition costs following a task cue-only by assigning participants to one of two groups depending on their mean duration of the blank interval. Subjects were assigned to one of two groups according to whether their individual mean duration of the blank interval was larger or smaller than the sample’s mean duration and this group variable was added as an additional between-subjects factor to the conventional ANOVA analyses in trial n. Hence, we performed an ANOVA on mean RTs and ERs in trial n depending on the within-subjects factors task set sequence (ABA, CBA) and n−2 trial type (task, task cue-only) as well as the between-subjects factors cue-task compatibility (compatible, incompatible) and blank group (mean blank duration > sample’s mean, mean blank duration < sample’s mean).

####  n−2 repetition costs depending on the response time in the preceding LDT

After equating the duration of the blank interval, one difference remained in the temporal sequence of trial triplets with a task cue-only compared to an executed task in trial n−2. RTs in the LDT were still roughly 70 ms shorter if it was preceded by a task cue-only compared to a task (see Supplementary Material B). Thus, the temporal distance between trial n−2 and onset of the task cue in trial n was not yet fully controlled. In order to test whether the presence of n−2 repetition costs following a task cue-only and the corresponding absence following an executed task in trial n−2 was a consequence of the shorter RT in the LDT in the former condition, we predicted RTs in trial n (for which n−2 repetition costs were observed in the present study) by the RT in the LDT in an explorative analysis. Using a similar LMM approach compared to the analyses predicting n−2 repetition costs by the duration of the blank interval, we predicted RTs in trial n by the RT in the LDT, the task set sequence, n−2 trial type and cue-task compatibility for both Experiment 1 and 2. Both models included random intercepts per participants, and the model for Experiment 1 further included random slopes for n−2 trial type and task set sequence, while for Experiment 2 only random slopes for n−2 trial type were included, as adding further random slopes resulted in poor model fit.

#### Effect course analysis and supplementary analyses

We performed the effect course analysis with the same specifications as in Experiment 1 with one exception. To foreshadow the results of Experiment 2, the *p*-value of the obtained cluster for compatible task cues was near the significance threshold (α = .05). Hence, to achieve a more precise estimation of the *p*-value of this cluster, we doubled the number of permutations and ran for this effect course analysis a total of 10,000 permutations. We performed the same supplementary analyses as in Experiment 1, which are reported in the Supplementary Material (A–D).

### Results

#### Switch costs in trial n−2

For the analysis of mean RTs in executed tasks in trial n−2 we neither observed a significant main effect of task sequence, *F*(1, 51) = 1.74, *p* = .193, nor of cue-task compatibility, *F*(1, 51) = 1.04, *p* = .314. Furthermore, the interaction of both factors did not reach significance, *F*(1, 51) = 3.34, *p* = .073. On a descriptive level, we observed small switch costs, i.e. when the task set of trial n of the previous trial triplet was repeated, for compatible cues (27 ms), but not for incompatible cues (−4 ms).

A corresponding analysis of mean ERs in trial n−2 revealed no significant effect. Neither any of the two main effects nor the interaction reached significance, all *F*s < 1.91, all *p*s > .173.

#### n−2* repetition costs*

**Fig. 4:**
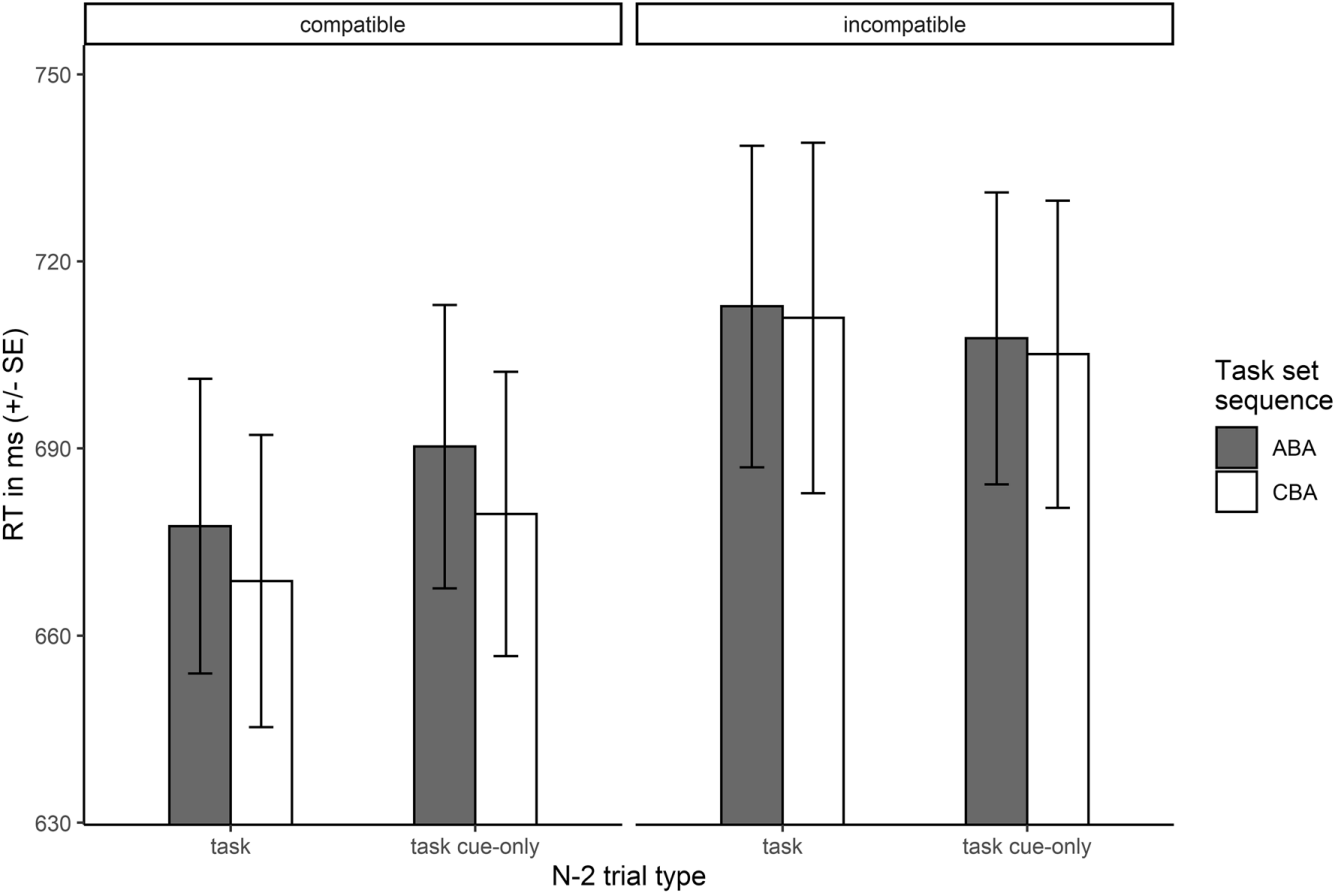
Response times in trial n in Experiment 2. Shown are mean RTs (± SE) in ms depending on n−2 trial type and task set sequence, separately for compatible and incompatible cues

A repeated-measures ANOVA on RTs in trial n revealed neither significant main effects of task set sequence, n−2 trial type, or cue-task compatibility, all *F*s < 1.52, all *p*s > .223, nor any significant interactions among these factors, all *F*s < 2.81, all *p*s > .099. Hence, in contrast to Experiment 1, RTs in the incompatible task cue condition (709 ms) did not significantly differ from that in the compatible task cue condition (679 ms). There was also no significant difference between task set sequences (ABA = 697 ms, CBA = 691 ms; *F*(1, 51) = 1.51, *p* = .224). Furthermore, and of particular relevance considering our research question, there were no significant n−2 repetition costs following a task cue-only (mean RT following a task cue-only in trial n−2: ABA = 699 ms, CBA = 692 ms; no significant interaction of task set sequence and n−2 trial type, *F*(1, 51) = 0.02, *p* = .883). Mean RTs in trial n are depicted in Fig. 4.

For the corresponding analysis of ERs, there was no significant effect at all, all *F*s < 0.19, all *p*s > .668. Mean ERs as well as mean RTs in trial n for Experiment 2 are reported in Table 3.

**Table 3:** Mean RTs and ERs in trial n in Experiment 2

Cue-task compatibility	n−2 trial type	Task set sequence	RT	ER
Compatible	Task	ABA	678 (24)	0.025 (0.005)
		CBA	669 (23)	0.029 (0.006)
	Task cue-only	ABA	690 (23)	0.026 (0.006)
		CBA	680 (23)	0.027 (0.006)
Incompatible	Task	ABA	713 (26)	0.029 (0.006)
		CBA	711 (28)	0.029 (0.007)
	Task cue-only	ABA	708 (23)	0.028 (0.005)
		CBA	705 (25)	0.028 (0.005)

Mean RTs are given in milliseconds, mean ERs in proportion of errors. Associated standard errors are shown in parentheses

#### n−2 repetition costs depending on the blank interval following a task cue-only

It could be hypothesized that the duration of the blank interval following a task-cue only predicts the size of subsequent n−2 repetition costs if task set activation following a task cue-only is thought to decay with time. To test this hypothesis, we performed two analyses. First, we predicted single-trial RTs and accuracies in trial n following a task cue-only in trial n−2 by the factors task set sequence, cue-task compatibility, and the duration of the blank interval (for a table showing all effects, see Supplementary Material G). For RTs, no effect except for the intercept reached significance, all other *|β|*s < 21.38, all *|t|*s < 1.41, all *p*s > .158.

A corresponding analysis on single-trial accuracies yielded no significant effect except for the intercept as well, all other *|β|*s < 0.21, all *|z|*s < 1.25, all *p*s > .213. Accordingly, these analyses did not reveal any significant influence of the duration of the blank interval on the size of n−2 repetition costs.

Second, we also tested whether between-subjects differences in the duration of the blank interval predicts the size of n−2 repetition costs by assigning participants to one of two groups according to whether their mean duration of the blank interval was larger or smaller than the samples’ mean. This variable (blank group) was added as additional between-subjects factor to the ANOVA analysis of mean RTs / ERs in trial n. For the analysis of RTs, there was only a main effect of blank group, *F*(1, 49) = 43.96, *p* < .001, reflecting longer RTs if the blank interval was larger (787 ms) compared to when it was smaller (619 ms) than the samples’ mean. This effect was not surprising as the blank duration following a task-cue only was calculated according to the RT of participants. No other effect including all interactions with the factor blank group reached significance, all *F*s < 2.73, all *p*s > .104.

For the corresponding ANOVA on mean ERs, we could not observe any significant effect. All *F*s < 2.11, all *p*s > .152.

####  n−2 repetition costs depending on the response time in the preceding LDT in Experiment 1 and Experiment 2

According to remaining differences in the LDT RT between n−2 trial types (see Supplementary Material B), we directly tested whether the RT in the LDT predicted the occurrence of RT n−2 repetition costs in the present study in an explorative analysis using LMMs for both Experiment 1 and Experiment 2. While the RT in the LDT generally predicted the RT in subsequent trial n, with shorter LDT RTs being associated with shorter trial n RTs, the LDT RT did not interact with the task set sequence in none of the two experiments (Experiment 1, *β* = 5.23, *t* = 1.16, *p* = .247; Experiment 2, *β* = 3.03, *t* = 0.63, *p* = .527). Furthermore, no higher-order interactions including task set sequence and LDT RT were significant (all *|β|*s < 12.17, all *|t|*s < 1.35, all *p*s > .181; for a full description of the LMM results, see Supplementary Material G).

#### Effect course analysis of n−2 repetition costs

**Fig. 5:**
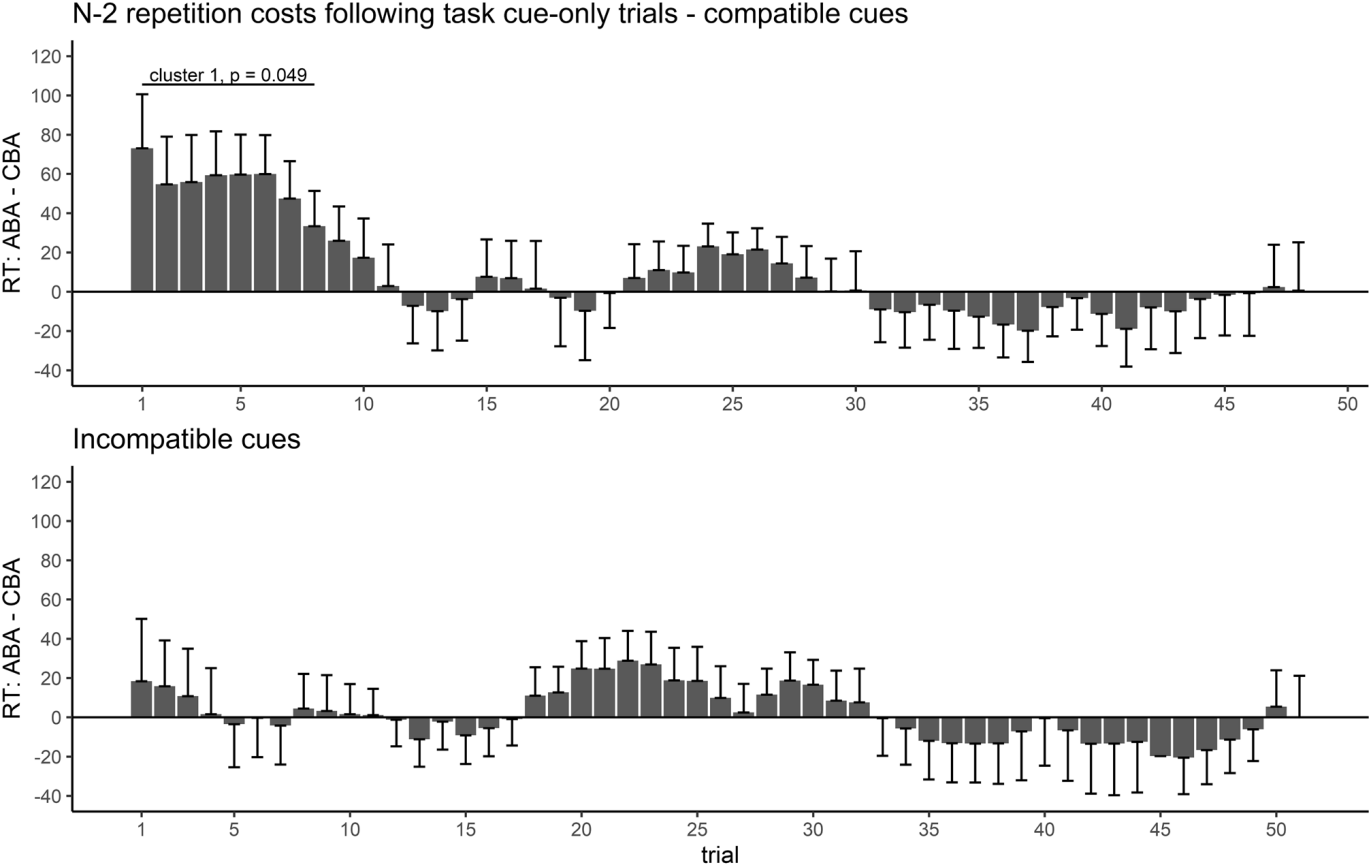
Development of response time n−2 repetition costs following a task cue-only in trial n−2 during the course of Experiment 2. Positive values indicate n−2 repetition costs, negative values n−2 repetition benefits. n−2 repetition costs were only observed for compatible cues and only at the beginning of the experiment

Effect course analyses on RT n−2 repetition costs following a task cue-only revealed a similar pattern as in Experiment 1, mainly for compatible cues, see Fig. 5. For compatible cues, n−2 repetition costs were maximal at the beginning of the experiment and decreased with practice. These n−2 repetition costs at the beginning of the experiment were associated with a significant cluster spanning from trials 1–8, cluster *T* = 20.18, *p* = .049. In contrast, for incompatible cues, no pronounced n−2 repetition costs were observed throughout the whole experiment at all, i.e. even descriptively, and no significant cluster was observed.

### Discussion

Regarding switch costs in executed tasks in trial n−2, we did not observe any significant effects on mean RTs and ERs. Additional drift-diffusion model analyses on combined RT and ER data revealed overall reliable switch costs independent of the cue task-compatibility conditions on the processing speed parameter, but repetition costs on the non-decisional time (see the Supplementary Material A). Hence, due to these contradictory findings in the drift-diffusion model analyses, switch costs as a treatment check were not unequivocally found in Experiment 2.

In contrast, regarding the main purpose of Experiment 2, i.e. to test whether n−2 repetition costs following a task cue-only can still be observed if the total duration of the task cue-only trial was comparable to that for task execution in trial n−2, the results were more clear-cut. While for average RTs and ERs no n−2 repetition costs (and no other effects) were observed, the effect course analyses on RTs revealed a similar pattern compared to Experiment 1. For compatible cues, n−2 repetition costs were maximal at the beginning of the experiment and vanished with practice. A significant cluster supported this presence of n−2 repetition costs at the beginning of the experiment. For incompatible cues, similar to Experiment 1, no significant cluster could be observed. While in Experiment 1, n−2 repetition costs for incompatible cues could be observed descriptively at the beginning of the experiment, which were reduced with practice, n−2 repetition costs for incompatible cues were not even found on a descriptive level in Experiment 2. Hence, lack of practice and cue-task compatibility remained the crucial factors for determining whether n−2 repetition costs can be obtained following a task cue-only. In contrast, the specific duration of the blank interval did not influence the pattern of n−2 repetition costs, which was demonstrated by analyses showing that the size of n−2 repetition costs neither depended on the duration of the blank interval in that trial triplet nor on the overall average duration of the blank interval. Accordingly, introducing a variable blank interval following a task cue-only did not change the pattern of n−2 repetition costs compared to Experiment 1, rendering it unlikely that a decay of task sets could explain the presence of n−2 repetition costs following a task cue-only and the absence of such costs following executed tasks.

While the pattern of n−2 repetition costs was similar in Experiments 1 and 2, i.e. irrespective of the duration of the blank interval, a more general difference remained between task cue-only and task n−2 trial types. The RT to the LDT was around approximately 70ms faster in both experiments when it was preceded by a task cue-only trial (but there were also more errors, suggesting a speed-accuracy tradeoff, see Supplementary Material B). Hence, it is still possible that task sets were inhibited to a similar degree following both task execution and task cue-only presentation, and only the longer RT in the following LDT caused the absence of n−2 repetition costs following task execution in trial n−2. Accordingly, instead of the temporal distance between trial n−2 and trial n−1 (cf. Gade & Koch, 2005), the distance between trial n−1 and trial n could be the crucial factor for the emergence of n−2 repetition costs in the present study (cf. Scheil & Kleinsorge, 2014b). However, while the RT in the LDT generally predicted the RT in subsequent trial n, with shorter LDT RTs being associated with shorter trial n RTs, the LDT RT did not significantly predict n−2 repetition costs in neither Experiment 1 nor Experiment 2. Thus, while we cannot definitively rule out that the different pattern of n−2 repetition costs following task execution and task cue-only trials depends on differences in the LDT RT, we would consider such an account unlikely given that the RT in the LDT did not emerge as a significant predictor of n−2 repetition costs.

To summarize, the similar result pattern in Experiment 2 compared to Experiment 1 is in agreement with our alternative explanation, suggesting that only cued, but not executed task sets require a larger amount of inhibition compared to executed task sets. We will return to this point in the “General discussion” section, where we also discuss more general performance differences between Experiment 1 and 2, which might be the consequence of introducing a variable blank interval.

## General discussion

In both Experiment 1 and 2, we observed in the effect course analyses n−2 repetition costs following a task cue-only, indicating that also merely prepared task sets are inhibited when switching to another task. More specifically, n−2 repetition costs were confined to compatible cues and to the beginning of the experiment, i.e. when tasks were not practiced intensively, highlighting the relevance of properties of the task cue and practice for the investigation of task set inhibition effects following a task cue-only. In contrast, following task execution, we did not observe n−2 repetition costs in neither of the two experiments. Hence, the presence of n−2 repetition costs following a task cue-only and the absence of such costs following task execution in trial n−2 cannot be readily explained by a decay of task set activation, as such a decay should have affected both task cue-only and task n−2 trial types similarly in Experiment 2. Hence, another account must explain the absence of n−2 repetition costs following task execution in the present experiments. However, there were more general performance differences between experiments, i.e. the lack of an effect of cue-task compatibility in Experiment 2, which might be the consequence of the introduction of a variable blank interval. We will return to these differences later and will first focus on the main purpose of the present study, the detection of task set inhibition effects following a task cue-only.

### Contribution of preparatory processes to task set inhibition effects

Combining the results of Experiment 1 and 2, the present study demonstrated that cue-induced task set activation is sufficient to produce an n−2 repetition cost. By presenting a task cue-only without following task in trial n−2, we argue that in contrast to previous work, this n−2 repetition cost occurring after task cue-only presentation can be clearly mapped to the processing of the task cue. Consequently, even for merely prepared task sets, there was a need to reconfigure these task sets in order to switch to a new task. This result pattern further suggests that task cue processing can be separated from task stimulus processing, as also the presentation of task cues in isolation affects task switching performance (Kleinsorge et al., 2005; Kleinsorge & Gajewski, 2004, 2006; Lenartowicz et al., 2011; Swainson et al., 2017). This need for a separate investigation of task cue processing is further highlighted by the absence of n−2 repetition costs following task execution in the present study. Hence, a task cue-only may induce even larger demands for task set inhibition compared to task execution.

In contrast, the absence of n−2 repetition costs for executed task sets in the present study might be explained by only low demands for inhibiting executed task sets. In both experiments, trial n−2 was either an executed task or a task cue-only, while trial n−1 was always an LDT, which was not explicitly cued and included different stimuli (letter strings comprising words or pseudowords) compared to the tasks in trials n−2 and n (which included pictures). n−2 repetition costs following a task in trial n−2 were shown to be reduced or even absent if aspects of trial n−1 differed from trials n−2 and n (Prosser et al., 2022; Scheil & Kleinsorge, 2023a, b; Sdoia et al., 2022). These previous studies suggest a critical influence of trial n−1 for the occurrence of n−2 repetition costs. Accordingly, our design—which was chosen to investigate whether our previous observations of an inhibition of task sets following a task cue-only in the context of a modulation of masked priming (Berger et al., 2022; Kiefer et al., 2019) also emerge in a task switching context—may not have been optimal for detecting n−2 repetition costs following task execution. Given the different stimulus format in the LDT compared to trials n−2 and n in the present study, the semantic and perceptual task sets could not be (erroneously) applied to the stimuli of the LDT. As a consequence, the interference between executed task sets in trial n−2 and the LDT was likely only low (cf. Vandierendonck et al., 2010). However, despite this supposedly low interference, there were no signs that participants could keep an executed task set active during the LDT, i.e. that the activation level of the task set previously triggered in trial n−2 remained constant throughout the LDT, i.e. from trial n−1 to trial n. Such an activation pattern should have resulted in repetition benefits, i.e. improved performance if the task set was repeated from trial n−2 to trial n. In contrast, we observed no pronounced differences between ABA and CBA sequences for executed tasks and n−2 repetition costs following task cue-only trials.

Notwithstanding the supposedly low interference between cued or executed task sets in trial n−2 and the LDT in trial n−1, response buttons overlapped between picture classification tasks and the LDT. Thus, there was at least competition on the level of response sets, which was shown to be a major determinant of n−2 repetition costs (Gade & Koch, 2007; Schuch & Koch, 2003). However, the present study cannot unambiguously determine whether this competition at the response set level resulted in task set inhibition following task cue-only trials. For example, recent research suggests that task switching may also occur in a more general, domain-independent manner, and therefore task sets may need to be updated regardless of the type of task change, i.e. any type of change may trigger a reconfiguration of task sets (cf. Kaiser et al., 2023; von Bastian & Druey, 2017). While we cannot decide whether competition within the response set or a change in the task set per se triggered task sets to be reconfigured in the present study, both accounts must incorporate an inhibition of (aspects of) the task set. Such an inhibitory mechanism is required to explain the occurrence of n−2 repetition costs following task cue-only trials. Nevertheless, since neither a competition at the level of the response set nor a more general updating demand of the required task set was sufficient to produce an n−2 repetition costs for executed tasks, a task cue-only must probably trigger a larger conflict with a new task, and the inhibitory mechanism to resolve this conflict must therefore be context-dependent to some extent.

### Origin of n−2 repetition costs following a task cue-only

The presence of n−2 repetition costs following a task cue-only and the absence of such costs following executed tasks in both experiments, i.e. even when the temporal sequence of both n−2 trial types was controlled, suggests that the need for task set inhibition is larger following a task cue-only trial compared to a task. Yet, the underlying processes driving this task set inhibition following task cue-only trials remain to be elucidated.

As a first consideration, we would consider it unlikely that the occurrence of n−2 repetition costs following the presentation of a task cue-only is the consequence of an inhibition of the cue representation. Previous work observed n−2 repetition costs even when a 2:1 cue-to-task mapping was implemented, suggesting that n−2 repetition costs are not bound to a repetition of the cue from trial n−2 to trial n but refer to the cued task set (Gade & Koch, 2008). Furthermore, in the present experiments, no cue was presented in trial n−1, i.e. there was no apparent conflict between cue representations, as two cues were never presented in direct succession. Accordingly, we consider it more likely that the n−2 repetition costs following a task cue-only were grounded in an inhibition of aspects of the task set.

Presumably, when participants processed the task cue, they already activated elements of the task set, likely including the required response set (especially when the cue-task relation was straightforward, see the next section). Moreover, we would consider participants—when preparing for a task in terms of processing the respective task cue—to also expect this task to occur (see Swainson et al., 2017, for a discussion of intentions to perform a task following a task cue-only). However, when then another task was presented (here the LDT), the occurrence of this different task compared to the intended-to-be-performed task resulted in a strong inhibition of the prepared task set. In contrast, after executing a task, participants also expected another task to occur, probably reducing the need for inhibiting the executed task set. Such a differential effect of task expectations for task cue-only and task n−2 trial types may constitute a similar effect compared to the increase of switch costs with a higher proportion of repetition trials (e.g., Bonnin et al., 2011; Dreisbach & Haider, 2006; Duthoo et al., 2012; Strivens et al., 2024), suggesting that it is more difficult to switch tasks if one expects tasks to repeat (for reviews, see Bugg & Crump, 2012; Dreisbach & Fröber, 2019). Regarding this line of reasoning, note that in the present study, task cues were followed by a task in 75% of all trials (trial n−2: 50%, trial n: 100%), rendering the lack of a task in task cue-only trials a distinct or salient event. This might induce especially high demands to inhibit task sets (for a similar argument for switch costs following a task cue-only, see Swainson et al., 2021). Hence, further studies could directly test the prediction that a higher proportion of task cue-only trials is related to a reduced inhibition of only cued, but not executed task sets.

Another aspect addresses the time point when this proposed task set inhibition occurs. Two competing accounts appear to be reasonable: First, the cued task set might be inhibited in terms of a “self-inhibition” (Gade et al., 2014; Houghton & Tipper, 1996) when the lack of the cued task is evident. Second, it might be inhibited during performance of the LDT due to a conflict between the cued task set and the task set of the LDT, in terms of a “lateral” inhibition process to resolve this conflict (Mayr & Keele, 2000). According to the susceptibility of n−2 repetition costs to manipulations of trial n−1 (e.g., Philipp et al., 2007; Schuch & Koch, 2003), n−2 repetition costs following executed tasks are mostly assumed to be grounded in the second mechanism (Koch et al., 2010). Regarding the present study, a self-inhibition mechanism seems equally plausible, as the absence of n−2 repetition costs following executed tasks renders the contribution of a lateral inhibition mechanism for the emergence of n−2 repetition costs less likely (at least for executed task sets). Such a self-inhibition mechanism could well take place before the response in trial n−1 is selected; it only requires participants to capture the absence of a task in trial n−2. However, this reasoning is clearly speculative, as we did not vary trial n−1 and only assessed inhibition in terms of after-effects on trial n.

### Influence of cue-task compatibility on cue-induced task set inhibition

The above-outlined processes resulting in n−2 repetition costs following a task cue-only seem to be more pronounced for compatible cues, as n−2 repetition costs were statistically confined to compatible cues as revealed by the effect course analysis. For incompatible cues, n−2 repetition costs following a task cue-only were only observed descriptively (Experiment 1) or lacked completely (Experiment 2). At a first glance, the observation of larger n−2 repetition costs for compatible cues compared to incompatible cues might seem counter-intuitive, as in task switching larger switch costs (Gade & Steinhauser, 2020; Jost et al., 2013) and n−2 repetition costs (Gade & Koch, 2014; Houghton et al., 2009) were usually observed for incompatible / less transparent task cues. However, our previous work (Kiefer et al., 2019) and a previous task switching study (Jost et al., 2017) indicated that more dominantly represented task sets are more strongly inhibited. Hence, the influence of cue-task compatibility / task cue transparency on the inhibition of task sets might be influenced by how the task (set) is mentally represented in contrast to conflicting tasks (sets). When switching between three different tasks with trivalent stimuli (i.e. each task can be applied on each stimulus), in which tasks probably strongly interfere with each other, compatible task cues might be especially helpful for task set activation and execution, as task sets can be more efficiently retrieved from memory (Logan & Schneider, 2006).^5^ In contrast, in a setting, in which not every task set can be applied on each stimulus like in the present work, task sets associated with compatible cues appear to produce a larger conflict with other task sets. This might be the consequence of a stronger and / or faster activation of cued task sets for compatible compared to incompatible task cues (Kiefer et al., 2019). Additionally, an increased expectation of the cued task for compatible cues could further add to this effect. The expectation of a task might be increased for compatible cues according to the more straightforward cue-task relation and an accordingly facilitated retrieval of task sets, thereby increasing inhibition effects when, unexpectedly, another task is presented. Such an influence might be especially pronounced when only little task practice is available, as indicated by a susceptibility of cue-induced task set inhibition effects to task practice, which we will discuss in the next section.

### Practice influences on cue-induced task set inhibition

N−2 repetition costs following a task cue-only for compatible cues were only reliable at the beginning of both experiments, reflected by a significant cluster, while disappearing later during the experiment. Hence, the assumed inhibition of task sets triggered by compatible cues appeared to vanish with longer experimental duration. Accordingly, both a lateral inhibition mechanism due to a conflict between cued task set and the task set of the LDT or a self-inhibition of cued task sets must have been reduced with practice. Previously, n−2 repetition costs following task execution were reduced with increasing practice (Grange et al., 2019; Grange & Juvina, 2015; Scheil, 2016), a pattern typical for many experimental effects (Miller, 2023). Moreover, such a reduction of n−2 repetition costs might reflect a more general account assuming more efficient (multi-tasking) processing as a function of practice (Koch et al., 2018). It appears reasonable that participants try to (at least implicitly) optimize their processing throughout an experimental session. Such an optimization process likely also decreases cue-induced task set inhibition effects in terms of reducing interference between task sets, as task sets become less vulnerable to interference of other task sets (Scheil & Kleinsorge, 2020, 2023a), called shielding of task sets (Dreisbach, 2012; Dreisbach & Haider, 2008). Alternatively, practice could even result in a qualitative shift of task cue processing in terms of a postponement of task set activation until the need of task execution is evident, i.e. the cued task is presented (cf. Berger et al., 2024). Such an argument would assume that participants reduced their engagement in task preparation during cue presentation with practice, as with increasing experimental duration participants more often experienced the lack of the (cued) task after task cue-only presentation. However, note that we previously observed increased task set inhibition effects following a task cue-only with practice, when even 50% of all trials were task cue-only trials (Berger et al., 2022). Hence, an alternative argument could be that cue-induced task set activation is strengthened with practice, and the strength of this task preparation in trial n is large enough to exceed any after-effects of trial n−2. In any case, the present study clearly indicates that task cue-induced processing is adapted within an experimental session.

### Effects of introducing a variable blank interval

While the results generally supported the above outlined postulated inhibition of task sets following a task cue-only in both experiments; Experiment 2 differed from Experiment 1 regarding the influence of cue-task compatibility on task performance in the semantic / perceptual classification tasks. For task performance in both trial n and trial n−2, on a descriptive level, mean RTs for compatible cues were remarkably longer in Experiment 2 compared to Experiment 1, while for incompatible cues RTs were slightly shorter in Experiment 2 compared to Experiment 1. Note that for both experiments, while being only significant in Experiment 1, RTs remained numerically longer for incompatible compared to compatible cues, similar to previous findings in the task switching literature, indicating a facilitated processing if cues are compatible / transparent (Jost et al., 2013; Kiesel et al., 2010). Furthermore, switch costs between trial n and trial n−2 of the next trial triplet were only significant in Experiment 1, suggesting an overall reduced robustness of effects in Experiment 2.

We can only speculate about the contribution of possible processes underlying the partially different results patterns in Experiments 1 and 2: In Experiment 2, the introduction of a variable blank interval might have introduced a temporal uncertainty to the participants. While there were also variable timing intervals in Experiment 1 according to the trial-to-trial varying RT, these variations were self-paced and thus probably subjectively more predictable. In contrast, an (both subjectively and objectively) unpredictability of trials in Experiment 2 due to the introduction of a randomly varying blank interval might have hampered task preparation or task execution processes.

The absence of significant RT differences between compatible and incompatible cues in Experiment 2 could be the result of another, counter-acting, mechanism, which may have facilitated performance for incompatible cues in Experiment 2. The introduction of a variable blank interval was accompanied by a general increase of the blank interval following a task cue-only. Probably, participants used this additionally available time for rehearsing the cue-task association, which should be especially helpful in the participant group with incompatible cues, for which the cue-task relation was less straightforward (cf. Logan & Schneider, 2006). For participants with incompatible cues, this benefit might have reduced the overall performance decrement due to the increased unpredictability of the experimental flow, resulting in less pronounced differences between compatible and incompatible cues in Experiment 2 (see also Supplementary Material B, which showed differences between cue-task compatibility groups in Experiment 2 to be also reduced for the LDT).

## Conclusions

The present study detected n−2 repetition costs following only cued, but not executed task sets. As stimulus- or task-related processes cannot explain such an n−2 repetition cost following a task cue-only, this effect can be best explained by an inhibition of cued task sets. Accordingly, the present study suggests that not only executed, but also merely cued task sets need to be reconfigured in order to switch to another, conflicting task. This cue-induced task set inhibition effect, however, was only observed for compatible cues with a straightforward cue-task association and only at the beginning of an experimental session. Our study thus highlights the significance of task cue properties and task practice as factors that moderate task set inhibition effects following a task cue-only.

## Supplementary Information

Below is the link to the electronic supplementary material.

Supplementary file1 (DOCX 147 KB)

## Data Availability

Data and analysis scripts for both Experiment 1 and Experiment 2 have been uploaded to a public repository and can be assessed at the following link: https://osf.io/ady8t/.

## Footnotes

1 Note that n−2 repetition costs are also called backward inhibition effect. For the sake of consistency, we will stick to the more operational term n−2 repetition costs.

2 Note that the different cue conditions were only called “compatible” and “incompatible” in the study of Berger et al. (2022). However, to facilitate comparison across studies, we re-formulated the results of the other studies in terms of the specification given by Berger et al. (2022).

3 This hypothesis was not explicitly pre-registered. We pre-registered the effect course analysis, but did not specify distinct hypotheses regarding its outcome. Hence, this hypothesis must be considered explorative.

4 Task sequence was collapsed across the semantic and perceptual task, i.e. we analyzed n-2 repetition costs across task sets. Note that this is a typical approach in the analysis of n-2 repetition costs.

5 While this process should affect the activation of a new task set, it does not need to necessarily affect the inhibition of a task set. However, even if task sets are more strongly inhibited for compatible task cues in the mentioned example, the more efficient retrieval of task sets could counteract a possibly stronger inhibition, resulting in overall lower n-2 repetition costs.
