# Supplementary material for: Inhibition of cued but not executed task sets depends on cue-task compatibility and practice
Source: Psychol Res. 2024 Jul 30;88(7):2036–58. doi: 10.1007/s00426-024-02013-z (PMC11450066; doi:10.1007/s00426-024-02013-z)
Supplement: Supplementary file 1 — Supplementary file1 (DOCX 147 KB) [file 426_2024_2013_MOESM1_ESM.docx]

Supplementary Material

Inhibition of cued but not executed task sets depends on

cue-task compatibility and practice

**Berger, Koch, & Kiefer**

**A: Drift-diffusion model analysis of task performance in trial n-2**

Description of drift-diffusion model analyses

To identify the underlying processes of task performance in trial n-2, we performed drift-diffusion model analyses (Ratcliff, 1978; Ratcliff & McKoon, 2008; Voss et al., 2013). Hierarchical, Bayesian drift-diffusion model analyses were performed with the toolbox *HDDM* (Wiecki et al., 2013). Drift-diffusion models were already applied in task switching (Ging-Jehli & Ratcliff, 2020; Schmitz & Voss, 2012, 2014). In line with our previous work (Berger et al., 2022) and as pre-registered, we analyzed the drift-diffusion model parameter drift rate *ν* and non-decision time *t0*. The drift rate represents the speed of information accumulation, and is usually associated with the ease of task-related processing. The non-decision time is thought to represent task-unrelated processes like visual stimulus processing and response execution.

For this and all following drift-diffusion model analyses, we estimated drift-diffusion models with the parameters *ν* and *t0* varying for the within-subjects factors in separate models for compatible and incompatible cues. For model estimation, correct and incorrect responses were defined as the upper and lower boundary of the decision process, respectively, and the starting point *z* was fixed at *a* / 2. 10,000 samples were drawn with a burn in of 3000 samples. Visual inspections of the posteriors and model traces indicated sufficient model fit for all models. After model estimation, we handed the estimated model parameters to a Bayesian repeated-measures ANOVA using the statistical software *JASP* (JASP Team, 2020). Bayes factors (BFs) for the evidence of an effect were interpreted using the guidelines provided in Wagenmakers et al. (2017).

For the analysis of task performance in trial n-2, these ANOVAs included the within-subjects factor task sequence (task repetition, task switch) and the between-subjects factor cue-task compatibility (compatible, incompatible cues).

Experiment 1

For drift rates, we observed extreme evidence for an effect of task sequence, *BF* = 151.9. Drift rates were on average larger for task repetitions (2.46) than task switches (2.26). Furthermore, there was extreme evidence for an effect of cue-task compatibility, *BF* = 9,382.1. Drift rates were larger for compatible (2.61) than incompatible cues (2.13). Concerning the interaction of both factors, there was modest evidence for an effect (*BF* = 3.9). Numerically, switch costs were present for both compatible (repetition = 2.64, switch = 2.57) and incompatible cues (repetition = 2.29, switch = 1.97), but larger for incompatible cues. The analysis of non-decision times revealed no evidence for the presence of any effect, all *BF*s < 1.2.

To sum up, drift-diffusion model analyses in Experiment 1 indicated an increased processing speed for task repetitions compared to task switches, i.e. processing was facilitated if the task repeated. This effect was observed for both cue-task compatibility conditions. Moreover, processing was generally facilitated for compatible task cues reflected by larger drift rates for compatible cues.

Experiment 2

For the analysis of task performance in trial n-2 in Experiment 2, there was extreme evidence for an effect of task sequence on drift rates, *BF* = 1629.3. Drift rates were on average larger for task repetitions (2.39) compared to task switches (2.18). Furthermore, there was strong evidence for an effect of cue-task compatibility (*BF* = 11.1, drift rate for compatible cues = 2.45, for incompatible cues = 2.14), but no evidence for an interaction of task sequence and cue-task compatibility (*BF* = 0.4). There appeared to be comparable switch costs for both compatible (repetition = 2.58, switch = 2.32) and incompatible cues (repetition = 2.22, switch = 2.06).

Concerning the analysis of the non-decision time, there was very strong evidence for an effect of task sequence (*BF* = 67.8), but no evidence for an effect of cue-task compatibility or the interaction of both factors (both *BF*s < 0.5). The effect of task sequence was reflected by a larger non-decision time for task repetitions (0.334) than task switches (0.319).

Taken together, the effect of task sequence on drift rates and the absence of sufficient evidence for an interaction of task sequence and cue-task compatibility indicated a processing benefit for task repetitions, for both cue-task compatibility groups. The difference between cue-task compatibility groups was smaller in Experiment 2 compared to Experiment 1, which was predominantly the result of a reduced drift rate for compatible cues between Experiment 2 and 1. Furthermore, and only in Experiment 2, the analysis of the non-decision time *t0* indicated a larger non-decision time for task repetitions compared to task switches, which is quite surprising. Previous work observed an absence of differences in *t0* between task repetitions and switches if preparation time was long (Schmitz & Voss, 2012, 2014), but usually not a repetition benefit. Taken the reversed task switch effects on drift rate *ν* and non-decision time *t0* together, drift diffusion modelling of Experiment 2 did not yield unequivocal switch costs, presumably due to the variable post-cue interval in the task cue-only conditions, which reduced predictability of trials. For a further discussion, we refer to the main text.

**B: Analyses of mean RTs and ERs in the LDT in trial n-1**

Design

For the analysis of mean RTs and ERs in the LDT in trial n-1, we performed repeated-measures ANOVAs with the within-subjects factor n-2 trial type (whether the LDT is preceded by a task or a task cue-only) and the between-subjects factor cue-task compatibility (compatible, incompatible). Hence, these analyses tested whether switching from a performed task or a task cue-only to the LDT is associated with different costs and whether such costs are modulated by cue-task compatibility.

Experiment 1

For the analysis of RTs, there was a significant effect of n-2 trial type, *F*(1, 51) = 124.30, *p* < .001, and cue-task compatibility, *F*(1, 51) = 4.54, *p* = .038. The interaction of both factors did not reach significance, *F*(1, 51) = 0.26, *p* = .612. As can be seen in *Figure S1*, *panel A*, RTs in the LDT were slower if it was preceded by a task in trial n-2 and if task cues were incompatible. For ERs, the pattern was quite different. Only the effect of n-2 trial type reached significance, *F*(1, 51) = 21.32, *p* < .001, while the effect of cue-task compatibility and the interaction of both factors did not reach significance (both *F*s < 0.67, both *p*s > 0.419), see *Figure S1, panel B*. However, in contrast to the analysis of RTs, ERs were larger if the LDT was preceded by a task cue-only compared to a task.

Taken together, these analyses suggested kind of a speed-accuracy tradeoff (Heitz, 2014). While responses in the LDT were faster, if it was preceded by a task cue-only, they were also more error prone. Possibly, as participants did not execute a task in the previous trial, and therefore did not have to switch tasks (only task sets), responses were speeded up following a task cue-only. Furthermore, the preparation for the cued, but not executed task (set) in trial n-2 may have led to confusion, with participants sometimes incorrectly trying to implement the previously cued task set, leading to increased errors in the LDT. As the LDT was not cued, the worse performance for incompatible cues might seem surprising in the first step, but could represent a carry-over effect in terms of increased demands of task execution and / or task cue processing for incompatible cues, resulting in increased demands for switching to the LDT.


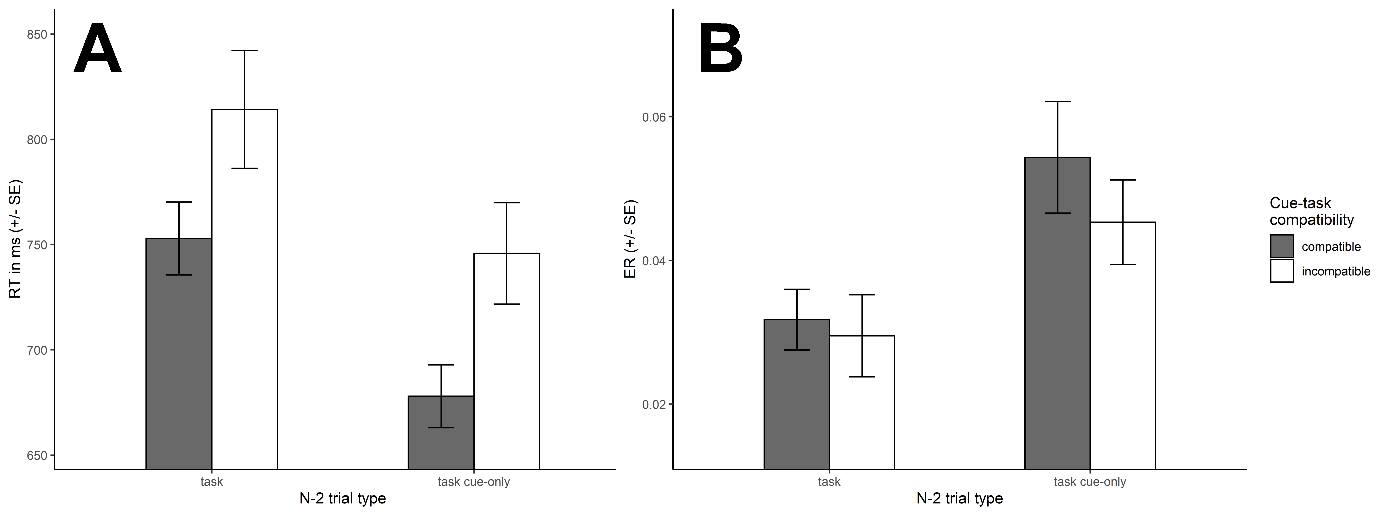


**Fig. S1** Response times (panel A) and error rates (panel B) in the lexical decision task (trial n-1) in Experiment 1. Shown are mean RTs and ERs (+- SE) in ms depending on n-2 trial type and cue-task compatibility.

Experiment 2

In Experiment 2, an ANOVA on RTs revealed a significant effect of n-2 trial type, *F*(1, 51) = 160.09, *p* < .001, reflecting slower RTs in the LDT if it was preceded by a task, see *Figure S2, panel A*. Neither the effect of cue-task compatibility nor the interaction of both factors reached significance (both *F*s < 0.50, both *p*s > .497). Concerning the analysis of ERs, there was a main effect of n-2 trial type, *F*(1, 51) = 13.77, *p* < .001, and cue-task compatibility, *F*(1, 51) = 8.28, *p* = .006. More errors were made, if the LDT was preceded by a task cue-only and for compatible cues. The interaction of n-2 trial type and cue-task compatibility failed to cross the significance threshold, *F*(1, 51) = 3.92, *p* = .053, but descriptively, the difference between n-2 trial types, showing more errors in the LDT following a task cue-only, was more pronounced for compatible cues, see *Figure S2, panel B*.

To sum up, the effect of n-2 trial type was comparable in Experiment 2 compared to Experiment 1; following a task cue-only, responses in the LDT were faster, but more error prone. However, the effect of cue-task compatibility changed between experiments. While RTs were slower for incompatible cues in Experiment 1, they were about the same size for both cue types in Experiment 2. In contrast, while also slightly observable in Experiment 1, but only on a descriptive level, participants with compatible cues conducted more errors compared to participants with incompatible cues in Experiment 2. To state possible reasons for this different result pattern concerning cue-task compatibility between experiments is difficult. Possibly, as more time was available following a task cue-only according to the increased blank interval in Experiment 2, participants could use this time for rehearsing the association between decision categories / tasks and task cues. Predominantly participants associated with incompatible cues, for which the relation of task cue and associated decision categories was less straightforward, should profit from such a rehearsal process, thereby enhancing performance in classification tasks, which should leave more cognitive resources available for performing the LDT. For such a possible influence, see also the less pronounced differences between cue-task compatibility groups in task performance in trial n and trial n-2 in Experiment 2 compared to Experiment 1 (*Supplementary Material A* and *D*).


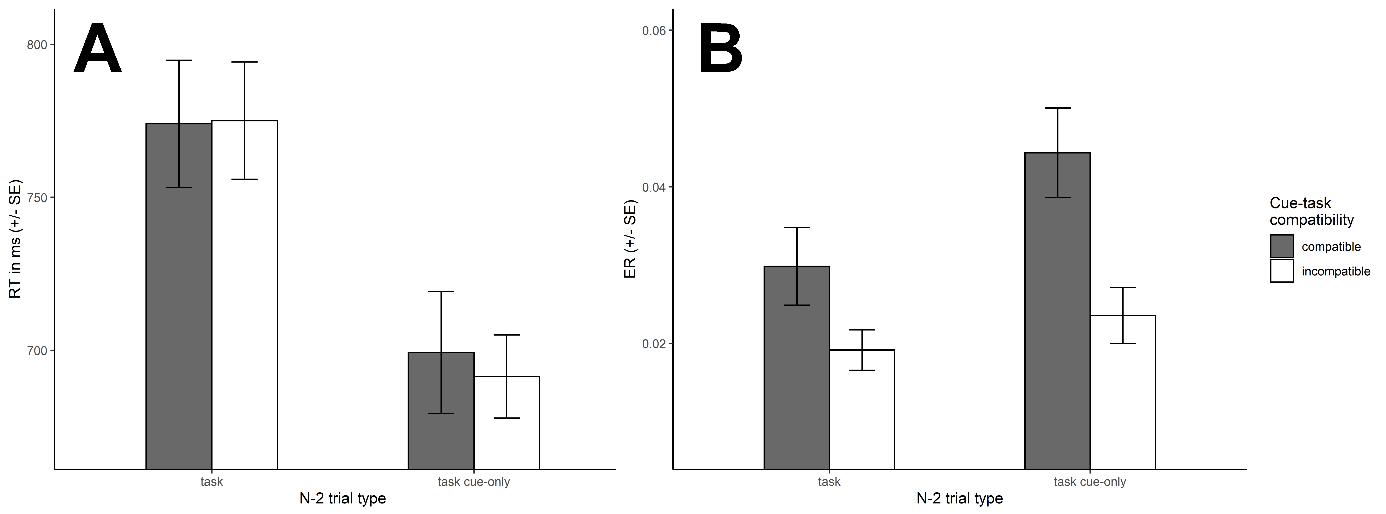


**Fig. S2** Response times (panel A) and error rates (panel B) in the lexical decision task (trial n-1) in Experiment 2.

**C: Drift-diffusion model analyses of the LDT in trial n-1**

Experiment 1

For performance in the LDT in Experiment 1, drift-diffusion model analyses revealed extreme evidence for an effect of n-2 trial type on drift rates, *BF* = 1113.4. There was no evidence for an effect of cue-task compatibility on drift rates (*BF* = 0.5), but moderate evidence for an interaction of cue-task compatibility and n-2 trial type, *BF* = 4.8. The difference between n-2 trial types in terms of larger drift rates, if the LDT was preceded by a task, was more pronounced for compatible cues (drift rate following a task = 2.78, following a task cue-only = 2.40) compared to incompatible cues (task = 2.57, task cue-only = 2.46). Concerning the analysis of the non-decision time, the Bayesian ANOVA revealed only an effect of n-2 trial type (extreme evidence, *BF* = 8.8 * e^11). For the effect of cue-task compatibility or the interaction of both factors, there was no convincing evidence, both *BF*s < 1.59. The effect of n-2 trial type was reflected by larger non-decision times following a task (0.486) compared to a task cue-only (0.397).

Hence, similar to the analysis of mean RTs and ERs (see *Supplementary Material B*), drift-diffusion model analyses indicated a kind of trade-off. Drift rates were larger in the LDT following an executed task, but in the same way, non-decision times were also elevated, indicating that task processing in the LDT was more efficient after a task, but also required more time to start and / or to be executed as response.

Experiment 2

For Experiment 2, corresponding drift-diffusion model analyses revealed strong evidence for an effect of n-2 trial type on drift rates, *BF* = 26.0, but no evidence for an effect of cue-task compatibility nor the interaction on drift rates, both *BF*s < 1.3. The effect of n-2 trial type was reflected by larger drift rates in the LDT following a task (2.87) compared to a task cue-only (2.70). The analysis of non-decision times revealed extreme evidence for an effect of n-2 trial type (*BF* = 4.2 * e^22), but not for the presence of the other two effects (both *BF*s < 1.0). Non decision times were larger following a task (0.473) compared to a task cue-only (0.385).

Taken together, drift-diffusion model analyses in the LDT in Experiment 2 mainly resembled the results of Experiment 1, with larger drift rates, but also elevated non-decision times following task execution in trial n-2.

**D: Drift-diffusion model analysis of performance in trial n**

Design

Drift-diffusion model analyses of task performance in trial n were performed on drift rates and non-decision times and used the same factors as the corresponding analyses on mean RTs and ERs. Hence, they included the within-subjects factors task set sequence (ABA, CBA) and n-2 trial type (task, task cue-only) as well as the between-subjects factor cue-task compatibility (compatible, incompatible).

Experiment 1

For Experiment 1, analyses of drift rates revealed extreme evidence for an effect of cue-task compatibility, *BF* = 207.9. Drift rates were larger for compatible (2.83) than incompatible cues (2.27). Furthermore, there was moderate evidence for an interaction of task set sequence and n-2 trial type, *BF* = 3.2. Following an executed task in trial n-2, drift rates were larger for sequences ABA (2.63) compared to CBA (2.50). In contrast, following a task cue-only, drift rates were smaller for sequences ABA (2.51) compared to CBA (2.55). Hence, considering drift rates, there was an n-2 repetition benefit, if the task set was executed in trial n-2, and a small repetition cost, if the task set was only cued, but not executed. For all other main effects and interactions, there was no convincing evidence, all *BF*s < 0.6. For the analysis of non-decision times, there was no evidence for any effect or interaction, all *BF*s < 0.9.

Taken together, drift-diffusion model analyses replicated the processing benefit for compatible compared to incompatible cues, which was already present in mean RTs. Considering n-2 repetition costs, it also showed a small repetition cost following a task cue-only. However, adding to the analyses on mean RTs and ERs, drift-diffusion model analyses could also reveal an n-2 repetition benefit for drift rates following task execution in trial n-2.

Experiment 2

Drift-diffusion model analyses of performance in trial n in Experiment 2 revealed no evidence for any main effect or interaction for drift rates, all *BF*s < 1.0. Similarly, for non-decision times, Bayes Factors also indicated no evidence for any main effect or interaction, all *BF*s < 1.4.

Accordingly, in contrast to Experiment 1, drift-diffusion model analyses in Experiment 2 neither indicated any evidence for an effect of cue-task compatibility, nor for an influence of the task set sequence, for neither n-2 trial type. To some degree, this is in line with a reduced difference on mean RTs for the two cue-task compatibility groups in Experiment 2, as well as the lack of any significant effect of task set sequence on mean RTs / ERs. In Experiment 2, an effect of task set sequence was only found in effect course analyses, and only for compatible cues at the beginning of the experiment (see the main text). Hence, drift-diffusion model analyses, which take into account the data of all available trials throughout the whole experiment, may have not been sensitive enough to detect such an influence being observable only at the beginning of the experiment comparable to the analysis of mean RTs.

**E: Tables for the ANOVA analyses for Experiment 1**

Switch costs in trial n-2

**Table S1** ANOVA results for RTs in trial n-2 in Experiment 1

| **Effect** | **Test statistic** | **p-value** | **partial η^2^** |
| --- | --- | --- | --- |
| Task sequence | F(1, 51) = 10.73 | 0.002 | 0.174 |
| Cue-task compatibility | F(1, 51) = 11.15 | 0.002 | 0.179 |
| Task sequence x cue-task compatibility | F(1, 51) = 10.61 | 0.002 | 0.172 |

**Table S2** ANOVA results for ERs in trial n-2 in Experiment 1

| **Effect** | **Test statistic** | **p-value** | **partial η^2^** |
| --- | --- | --- | --- |
| Task sequence | F(1, 51) = 2.91 | 0.094 | 0.054 |
| Cue-task compatibility | F(1, 51) = 1.93 | 0.171 | 0.036 |
| Task sequence x cue-task compatibility | F(1, 51) = 0.03 | 0.862 | ≈ 0 |

N-2 repetition costs

**Table S3** ANOVA results for RTs in trial n in Experiment 1

| **Effect** | **Test statistic** | **p-value** | **partial η^2^** |
| --- | --- | --- | --- |
| Task set sequence | F(1, 51) = 2.36 | 0.130 | 0.044 |
| N-2 trial type | F(1, 51) = 1.20 | 0.279 | 0.023 |
| Cue-task compatibility | F(1, 51) = 12.44 | < 0.001 | 0.196 |
| Task set sequence x cue-task compatibility | F(1, 51) = 0.03 | 0.864 | ≈ 0 |
| N-2 trial type x cue-task compatibility | F(1, 51) = 0.44 | 0.509 | 0.009 |
| Task set sequence x n-2 trial type | F(1, 51) = 4.09 | 0.048 | 0.074 |
| Task set sequence x n-2 trial type x cue-task compatibility | F(1, 51) = 0.68 | 0.415 | 0.013 |

**Table S4** ANOVA results for ERs in trial n in Experiment 1

| **Effect** | **Test statistic** | **p-value** | **partial η^2^** |
| --- | --- | --- | --- |
| Task set sequence | F(1, 51) ≈ 0 | 0.961 | ≈ 0 |
| N-2 trial type | F(1, 51) = 2.58 | 0.114 | 0.048 |
| Cue-task compatibility | F(1, 51) = 0.69 | 0.411 | 0.013 |
| Task set sequence x cue-task compatibility | F(1, 51) = 0.17 | 0.683 | 0.003 |
| N-2 trial type x cue-task compatibility | F(1, 51) = 0.75 | 0.391 | 0.014 |
| Task set sequence x n-2 trial type | F(1, 51) = 0.03 | 0.873 | ≈ 0 |
| Task set sequence x n-2 trial type x cue-task compatibility | F(1, 51) = 2.00 | 0.163 | 0.038 |

LDT

**Table S5** ANOVA results for RTs in trial n-1 in Experiment 1

| **Effect** | **Test statistic** | **p-value** | **partial η^2^** |
| --- | --- | --- | --- |
| N-2 trial type | F(1, 51) = 124.30 | < 0.001 | 0.709 |
| Cue-task compatibility | F(1, 51) = 4.54 | 0.038 | 0.082 |
| N-2 trial type x cue-task compatibility | F(1, 51) = 0.26 | 0.612 | 0.005 |

**Table S6** ANOVA results for ERs in trial n-1 in Experiment 1

| **Effect** | **Test statistic** | **p-value** | **partial η^2^** |
| --- | --- | --- | --- |
| N-2 trial type | F(1, 51) = 21.32 | < 0.001 | 0.295 |
| Cue-task compatibility | F(1, 51) = 0.58 | 0.451 | 0.011 |
| N-2 trial type x cue-task compatibility | F(1, 51) = 0.66 | 0.420 | 0.013 |

**F: Tables for the ANOVA analyses for Experiment 2**

Switch costs in trial n-2

**Table S7** ANOVA results for RTs in trial n-2 in Experiment 2

| **Effect** | **Test statistic** | **p-value** | **partial η^2^** |
| --- | --- | --- | --- |
| Task sequence | F(1, 51) = 1.74 | 0.193 | 0.033 |
| Cue-task compatibility | F(1, 51) = 1.04 | 0.314 | 0.020 |
| Task sequence x cue-task compatibility | F(1, 51) = 3.34 | 0.073 | 0.062 |

**Table S8** ANOVA results for ERs in trial n-2 in experiment 2

| **Effect** | **Test statistic** | **p-value** | **partial η^2^** |
| --- | --- | --- | --- |
| Task sequence | F(1, 51) = 0.45 | 0.506 | 0.009 |
| Cue-task compatibility | F(1, 51) = 1.90 | 0.174 | 0.036 |
| Task sequence x cue-task compatibility | F(1, 51) = 0.54 | 0.467 | 0.010 |

N-2 repetition costs

**Table S9** ANOVA results for RTs in trial n in Experiment 2

| **Effect** | **Test statistic** | **p-value** | **partial η^2^** |
| --- | --- | --- | --- |
| Task set sequence | F(1, 51) = 1.51 | 0.224 | 0.029 |
| N-2 trial type | F(1, 51) = 0.38 | 0.543 | 0.007 |
| Cue-task compatibility | F(1, 51) = 0.80 | 0.376 | 0.015 |
| Task set sequence x cue-task compatibility | F(1, 51) = 0.61 | 0.438 | 0.012 |
| N-2 trial type x cue-task compatibility | F(1, 51) = 2.80 | 0.100 | 0.052 |
| Task set sequence x n-2 trial type | F(1, 51) = 0.02 | 0.883 | ≈ 0 |
| Task set sequence x n-2 trial type x cue-task compatibility | F(1, 51) = 0.01 | 0.943 | ≈ 0 |

**Table S10** ANOVA results for ERs in trial n in Experiment 2

| **Effect** | **Test statistic** | **p-value** | **partial η^2^** |
| --- | --- | --- | --- |
| Task set sequence | F(1, 51) = 0.14 | 0.707 | 0.003 |
| N-2 trial type | F(1, 51) = 0.19 | 0.669 | 0.004 |
| Cue-task compatibility | F(1, 51) = 0.11 | 0.744 | 0.002 |
| Task set sequence x cue-task compatibility | F(1, 51) = 0.14 | 0.714 | 0.003 |
| N-2 trial type x cue-task compatibility | F(1, 51) = 0.02 | 0.901 | ≈ 0 |
| Task set sequence x n-2 trial type | F(1, 51) = 0.05 | 0.831 | ≈ 0 |
| Task set sequence x n-2 trial type x cue-task compatibility | F(1, 51) = 0.05 | 0.826 | ≈ 0 |

N-2 repetition costs including blank group

**Table S11** ANOVA results for RTs in trial n in Experiment 2 including the blank group variable

| **Effect** | **Test statistic** | **p-value** | **partial η^2^** |
| --- | --- | --- | --- |
| Task set sequence | F(1, 49) = 1.55 | 0.219 | 0.031 |
| N-2 trial type | F(1, 49) = 0.38 | 0.542 | 0.008 |
| Cue-task compatibility | F(1, 49) = 0.34 | 0.562 | 0.007 |
| Blank group | F(1, 49) = 43.96 | < 0.001 | 0.473 |
| Task set sequence x cue-task compatibility | F(1, 49) = 0.63 | 0.430 | 0.013 |
| Task set sequence x blank group | F(1, 49) = 1.39 | 0.244 | 0.028 |
| Task set sequence x n-2 trial type | F(1, 49) ≈ 0 | 0.966 | ≈ 0 |
| N-2 trial type x cue-task compatibility | F(1, 49) = 2.73 | 0.105 | 0.053 |
| N-2 trial type x blank group | F(1, 49) = 0.22 | 0.639 | 0.005 |
| Cue-task compatibility x blank group | F(1, 49) = 0.24 | 0.628 | 0.005 |
| Task set sequence x cue-task compatibility x blank group | F(1, 49) = 1.69 | 0.200 | 0.033 |
| Task set sequence x n-2 trial type x cue-task compatibility | F(1, 49) ≈ 0 | 0.976 | ≈ 0 |
| Task set sequence x n-2 trial type x blank group | F(1, 49) = 0.28 | 0.597 | 0.006 |
| N-2 trial type x cue-task compatibility x blank group | F(1, 49) = 0.40 | 0.530 | 0.008 |
| Task set sequence x n-2 trial type x cue-task compatibility x blank group | F(1, 49) = 0.23 | 0.635 | 0.005 |

**Table S12** ANOVA results for ERs in trial n in Experiment 2 including the blank group variable

| **Effect** | **Test statistic** | **p-value** | **partial η^2^** |
| --- | --- | --- | --- |
| Task set sequence | F(1, 49) = 0.32 | 0.572 | 0.007 |
| N-2 trial type | F(1, 49) = 0.32 | 0.575 | 0.006 |
| Cue-task compatibility | F(1, 49) = 0.09 | 0.770 | 0.002 |
| Blank group | F(1, 49) = 0.29 | 0.591 | 0.006 |
| Task set sequence x cue-task compatibility | F(1, 49) = 0.31 | 0.579 | 0.006 |
| Task set sequence x blank group | F(1, 49) = 2.11 | 0.153 | 0.041 |
| Task set sequence x n-2 trial type | F(1, 49) = 0.10 | 0.757 | 0.002 |
| N-2 trial type x cue-task compatibility | F(1, 49) ≈ 0 | 0.983 | ≈ 0 |
| N-2 trial type x blank group | F(1, 49) = 0.68 | 0.412 | 0.014 |
| Cue-task compatibility x blank group | F(1, 49) = 0.08 | 0.778 | 0.002 |
| Task set sequence x cue-task compatibility x blank group | F(1, 49) = 0.23 | 0.635 | 0.005 |
| Task set sequence x n-2 trial type x cue-task compatibility | F(1, 49) = 0.10 | 0.753 | 0.002 |
| Task set sequence x n-2 trial type x blank group | F(1, 49) = 1.36 | 0.250 | 0.027 |
| N-2 trial type x cue-task compatibility x blank group | F(1, 49) = 0.37 | 0.545 | 0.008 |
| Task set sequence x n-2 trial type x cue-task compatibility x blank group | F(1, 49) = 0.03 | 0.869 | ≈ 0 |

LDT

**Table S13** ANOVA results for RTs in trial n-1 in Experiment 2

| **Effect** | **Test statistic** | **p-value** | **partial η^2^** |
| --- | --- | --- | --- |
| N-2 trial type | F(1, 51) = 160.09 | < 0.001 | 0.758 |
| Cue-task compatibility | F(1, 51) = 0.02 | 0.894 | ≈ 0 |
| N-2 trial type x cue-task compatibility | F(1, 51) = 0.50 | 0.484 | 0.010 |

**Table S14** ANOVA results for ERs in trial n-1 in Experiment 2

| **Effect** | **Test statistic** | **p-value** | **partial η^2^** |
| --- | --- | --- | --- |
| N-2 trial type | F(1, 51) = 13.77 | < 0.001 | 0.213 |
| Cue-task compatibility | F(1, 51) = 8.28 | 0.006 | 0.140 |
| N-2 trial type x cue-task compatibility | F(1, 51) = 3.92 | 0.053 | 0.071 |

**G: Tables for the LMM analyses for Experiment 1 and 2**

N-2 repetition costs depending on the duration of the blank interval in Experiment 2

**Table S15** LMM results for RTs in trial n in Experiment 2 depending on the duration of the blank interval in trial n-2

| **Fixed effects** | **beta** | **SE** | **t** | **p-value** |
| --- | --- | --- | --- | --- |
| Intercept | 695.81 | 16.3 | 42.60 | < 0.001 |
| Task set sequence | 6.25 | 6.5 | 0.97 | 0.334 |
| Cue-task compatibility | -21.37 | 32.7 | -0.65 | 0.516 |
| Blank duration (z-standardized) | 0.97 | 3.8 | 0.26 | 0.798 |
| Task set sequence x cue-task compatibility | 7.31 | 12.9 | 0.57 | 0.572 |
| Task set sequence x blank duration | 4.67 | 6.5 | -0.72 | 0.474 |
| Cue-task compatibility x blank duration | -8.17 | 7.6 | -1.08 | 0.285 |
| Task set sequence x cue-task compatibility x blank duration | 18.39 | 13.1 | 1.41 | 0.159 |

Note: Only trial triplets with a task cue-only in trial n-2 were included

**Table S16** LMM results for response correctness in trial n in Experiment 2 depending on the duration of the blank interval in trial n-2

| **Fixed effects** | **beta** | **SE** | **z** | **p-value** |
| --- | --- | --- | --- | --- |
| Intercept | 3.86 | 0.2 | 25.68 | < 0.001 |
| Task set sequence | 0.03 | 0.2 | 0.17 | 0.862 |
| Cue-task compatibility | 0.08 | 0.3 | 0.28 | 0.778 |
| Blank duration (z-standardized) | 0.02 | 0.1 | 0.20 | 0.841 |
| Task set sequence x cue-task compatibility | 0.04 | 0.3 | 0.12 | 0.908 |
| Task set sequence x blank duration | 0.02 | 0.2 | 0.12 | 0.902 |
| Cue-task compatibility x blank duration | -0.20 | 0.2 | -1.24 | 0.214 |
| Task set sequence x cue-task compatibility x blank duration | -0.09 | 0.3 | -0.28 | 0.783 |

Note: Only trial triplets with a task cue-only in trial n-2 were included

N-2 repetition costs depending on the RT in the LDT in trial n-1

**Table S17** LMM results for RTs in trial n in Experiment 1 depending on the RT in the LDT in trial n-1

| **Fixed effects** | **beta** | **SE** | **t** | **p-value** |
| --- | --- | --- | --- | --- |
| Intercept | 669.32 | 17.6 | 38.01 | < 0.001 |
| Task set sequence | 7.22 | 5.5 | 1.31 | 0.195 |
| N-2 trial type | 15.51 | 5.8 | 2.69 | 0.010 |
| Cue-task compatibility | -124.54 | 35.2 | -3.54 | < 0.001 |
| RT LDT (z-standardized) | 21.73 | 2.3 | 9.62 | < 0.001 |
| Task set sequence x n-2 trial type | 24.31 | 9.0 | 2.71 | 0.007 |
| Task set sequence x cue-task compatibility | 1.91 | 11.0 | 0.17 | 0.863 |
| N-2 trial type x cue-task compatibility | 5.60 | 11.6 | 0.48 | 0.630 |
| Task set sequence x RT LDT | 5.23 | 4.5 | 1.16 | 0.247 |
| N-2 trial type x RT LDT | 2.34 | 4.5 | 0.52 | 0.606 |
| Cue-task compatibility x RT LDT | -9.88 | 4.5 | -2.19 | 0.029 |
| Task set sequence x n-2 trial type x cue-task compatibility | -12.16 | 17.9 | -0.68 | 0.497 |
| Task set sequence x n-2 trial type x RT LDT | -12.09 | 9.1 | -1.34 | 0.182 |
| Task set sequence x cue-task compatibility x RT LDT | 10.46 | 9.0 | 1.16 | 0.247 |
| N-2 trial type x cue-task compatibility x RT LDT | -3.43 | 9.1 | -0.38 | 0.706 |
| Task set sequence x n-2 trial type x cue-task compatibility x RT LDT | 7.11 | 18.1 | 0.39 | 0.694 |

**Table S18** LMM results for RTs in trial n in Experiment 2 depending on the RT in the LDT in trial n-1

| **Fixed effects** | **beta** | **SE** | **t** | **p-value** |
| --- | --- | --- | --- | --- |
| Intercept | 692.97 | 16.9 | 40.95 | < 0.001 |
| Task set sequence | 7.23 | 4.8 | 1.52 | 0.128 |
| N-2 trial type | 13.52 | 5.5 | 2.48 | 0.016 |
| Cue-task compatibility | -30.12 | 33.8 | -0.89 | 0.378 |
| RT LDT (z-standardized) | 20.90 | 2.4 | 8.77 | < 0.001 |
| Task set sequence x n-2 trial type | 3.28 | 9.5 | 0.35 | 0.730 |
| Task set sequence x cue-task compatibility | 7.49 | 9.5 | 0.79 | 0.431 |
| N-2 trial type x cue-task compatibility | 19.28 | 10.9 | 1.76 | 0.083 |
| Task set sequence x RT LDT | 3.03 | 4.8 | 0.63 | 0.527 |
| N-2 trial type x RT LDT | -8.88 | 4.8 | -1.85 | 0.064 |
| Cue-task compatibility x RT LDT | 4.46 | 4.8 | 0.94 | 0.350 |
| Task set sequence x n-2 trial type x cue-task compatibility | 0.07 | 19.0 | ≈ 0 | 0.997 |
| Task set sequence x n-2 trial type x RT LDT | 11.17 | 9.6 | 1.17 | 0.243 |
| Task set sequence x cue-task compatibility x RT LDT | -2.73 | 9.6 | -0.29 | 0.775 |
| N-2 trial type x cue-task compatibility x RT LDT | -2.24 | 9.6 | -0.23 | 0.816 |
| Task set sequence x n-2 trial type x cue-task compatibility x RT LDT | -1.70 | 19.1 | -0.09 | 0.929 |

Supplementary references

Berger, A., Kunde, W., & Kiefer, M. (2022). Task cue influences on lexical decision performance and masked semantic priming effects: The role of cue-task compatibility. *Attention, Perception, and Psychophysics*, *84*(8), 2684–2701. https://doi.org/10.3758/S13414-022-02568-2/FIGURES/3

Ging-Jehli, N. R., & Ratcliff, R. (2020). Effects of aging in a task-switch paradigm with the diffusion decision model. *Psychology and Aging*, *35*(6), 850–865. https://doi.org/10.1037/PAG0000562

Heitz, R. P. (2014). The speed-accuracy tradeoff: History, physiology, methodology, and behavior. *Frontiers in Neuroscience*, *8*(8 JUN), 86875. https://doi.org/10.3389/FNINS.2014.00150/BIBTEX

JASP Team. (2020). *JASP (Version 0.14.1)[Computer software]*. https://jasp-stats.org/

Ratcliff, R. (1978). A theory of memory retrieval. *Psychological Review*, *85*(2), 59–108. https://doi.org/10.1037/0033-295X.85.2.59

Ratcliff, R., & McKoon, G. (2008). The diffusion decision model: Theory and data for two-choice decision tasks. *Neural Computation*, *20*(4), 873–922. https://doi.org/10.1162/NECO.2008.12-06-420

Schmitz, F., & Voss, A. (2012). Decomposing task-switching costs with the diffusion model. *Journal of Experimental Psychology: Human Perception and Performance*, *38*(1), 222–250. https://doi.org/10.1037/A0026003

Schmitz, F., & Voss, A. (2014). Components of task switching: A closer look at task switching and cue switching. *Acta Psychologica*, *151*, 184–196. https://doi.org/10.1016/J.ACTPSY.2014.06.009

Voss, A., Nagler, M., & Lerche, V. (2013). Diffusion models in experimental psychology: a practical introduction. *Experimental Psychology*, *60*(6), 385–402. https://doi.org/10.1027/1618-3169/A000218

Wagenmakers, E.-J., Love, J., Marsman, M., Jamil, T., Ly, A., Verhagen, J., Selker, R., Gronau, Q. F., Dropmann, D., Boutin, B., Meerhoff, F., Knight, P., Raj, A., van Kesteren, E.-J., van Doorn, J., Šmíra, M., Epskamp, S., Etz, A., Matzke, D., … Morey, R. D. (2017). Bayesian inference for psychology. Part II: Example applications with JASP. *Psychonomic Bulletin & Review 2017 25:1*, *25*(1), 58–76. https://doi.org/10.3758/S13423-017-1323-7

Wiecki, T. V, Sofer, I., & Frank, M. J. (2013). HDDM: Hierarchical Bayesian estimation of the Drift-Diffusion Model in Python. *Frontiers in Neuroinformatics*, *0*(JULY 2013), 14. https://doi.org/10.3389/FNINF.2013.00014
